# CD4^+^ T Cells Are Key to Shaping a Protective Humoral Immunity in Primary Dengue 2 Virus Infection: Implications for Rational Vaccine Design

**DOI:** 10.3390/vaccines13111103

**Published:** 2025-10-29

**Authors:** Angel E. Miranda-Santiago, Crisanta Serrano-Collazo, Lorna A. Cruz, Sandra Henein, Laura Alvarez, Teresa Arana, Jorge L. Sánchez-Bibiloni, Melween I. Martinez, Chiara Roman, Armando G. Burgos, Marcos J. Ramos-Benitez, Lourdes M. Caro-Rivera, James D. Brien, Amelia K. Pinto, Aravinda M. de Silva, Carlos A. Sariol

**Affiliations:** 1 Department of Microbiology and Medical Zoology, University of Puerto Rico-Medical Sciences Campus, San Juan, PR 00936-5067, USA; angel.miranda3@upr.edu (A.E.M.-S.); crisanta.serrano@upr.edu (C.S.-C.); lorna.cruz@upr.edu (L.A.C.); laura.alvarez1@upr.edu (L.A.); teresa.arana@upr.edu (T.A.); jorge.sanchez15@upr.edu (J.L.S.-B.); 2 Unit of Comparative Medicine, Caribbean Primate Research Center, University of Puerto Rico-Medical Sciences Campus, San Juan, PR 00936-5067, USA; melween.martinez@upr.edu (M.I.M.); chiara.roman@upr.edu (C.R.); armando.burgos@upr.edu (A.G.B.); 3 Department of Microbiology and Immunology, University of North Carolina Chapel Hill, Chapel Hill, NC 27599-0001, USA; sandra_henein@med.unc.edu (S.H.); aravinda_desilva@med.unc.edu (A.M.d.S.); 4 Basic Science Department, Microbiology, Ponce Health Sciences University, Ponce, PR 00732, USA; mjramos@psm.edu (M.J.R.-B.); lcaro23@stu.psm.edu (L.M.C.-R.); 5 Department of Microbiology, Immunology, and Molecular Genetics, College of Medicine, University of Kentucky, Lexington, KY 40506, USA; j.brien@uky.edu (J.D.B.); amelia.pinto@uky.edu (A.K.P.); 6 Department of Internal Medicine, University of Puerto Rico-Medical Sciences Campus, San Juan, PR 00936-5067, USA

**Keywords:** flavivirus, dengue, zika, cross-reactivity, rhesus, CD4^+^ T cell depletion, humoral response priming

## Abstract

**Background:** Understanding the immune mechanisms that differentiate protective from pathogenic responses during dengue virus (DENV) infection is critical for effective vaccine development. **Objective:** To investigate how CD4^+^ T cell depletion alters viral control and the humoral immune response during primary DENV2 infection in a non-human primate (NHP) model. **Methods:** Rhesus macaques were depleted of CD4^+^ T cells prior to DENV2 infection. Viral kinetics, B cell activation, antibody specificity, and functional outcomes were evaluated longitudinally, including cross-reactivity and antibody-dependent enhancement (ADE) potential. **Results:** CD4^+^ T cells were essential for early viral clearance and the generation of robust, type-specific neutralizing antibodies. In their absence, animals exhibited early non-specific polyclonal B cell activation, delayed isotype switching, and an expanded repertoire of cross-reactive antibodies to DENV and Zika virus (ZIKV), with diminished neutralizing capacity. CD4-depleted macaques also showed increased ADE potential, particularly against ZIKV, and elevated anti-NS1 IgG titers that persisted one-year post-infection. **Conclusion:** CD4^+^ T cells play a critical role in orchestrating effective, durable, and type-specific antibody responses during primary DENV infection. Their absence leads to delayed antibody maturation, greater cross-reactivity, and higher ADE potential. These findings emphasize the need for DENV and ZIKV vaccines to include CD4^+^ T cell epitopes that promote high-quality, type-specific antibody responses and minimize ADE risk.

## 1. Introduction

Despite existing vaccine efforts (e.g., Dengvaxia, TAK-003), concerns remain over non-neutralizing antibody responses and antibody-dependent enhancement (ADE). Elucidating the cellular drivers of protective humoral immunity during primary dengue virus (DENV) infections is thus essential for improved vaccine designs. Our work has implications for understanding the increased severity in the context of vaccines already deployed and real-world data from a mechanistic point of view. In both vaccines, the unbalanced replication of DENV serotypes has been documented [1,2,3,4,5,6,7], implying a potential unbalanced priming of CD4^+^ T cells during vaccination. Vaccines such as the ones aforementioned have shown varied efficacy, depending on the infecting serotype, and are a topic of ongoing active debate. In the case of Dengvaxia, it only showed better efficacy in DENV seropositive individuals [8]. Post-hoc pooled analysis from CYD14, CYD15, and long-term follow-up trials demonstrated increased risk of hospitalized and severe dengue in baseline-seronegative vaccinees upon subsequent natural infection [9]. Also in Latin America, a phase-3 trial showed good efficacy overall, but later analyses revealed differential protection varied with baseline serostatus. However, this dengue vaccine was efficacious against Virological Confirmed Dengue (VCD) and severe cases and led to fewer hospitalizations for VCD [5]. On the other hand, a long-term safety and efficacy study of TAK-003 through 4.5 years of follow-up, the vaccine showed robust protection overall, highest against DENV2, with stronger efficacy in seropositive individuals [7]. However, in another long-term safety and efficacy study also of 4.5 years of follow-up, no safety signal of increased severe dengue in seronegative individuals was seen, with modest waning efficacy for DENV-3 [10]. An independent expert commentary highlights the absence of ADE signal; however, it draws attention to the need for extended monitoring [11].

DENV and Zika virus (ZIKV) are globally significant mosquito-borne flaviviruses that continue to pose increasing public health challenges, particularly in tropical and subtropical regions [12,13,14,15,16]. DENV alone accounts for an estimated 390–500 million infections annually, with a substantial burden of morbidity and mortality, particularly in cases progressing to severe manifestations such as dengue hemorrhagic fever (DHF) and dengue shock syndrome (DSS) [17,18,19]. Factors such as rapid urbanization, ecological and climatic shifts play pivotal roles in both the expanding range of the mosquito (*Aedes aegypti*) vector and in creating more breeding sites close to highly populated areas.

Despite substantial advances in our understanding of DENV biology and immunology, the immune correlates that distinguish protective from pathogenic responses during primary infections remain poorly defined, complicating vaccine development efforts. One of the most controversial and complex aspects of flavivirus immunity is the dual role of antibodies. Type-specific (TS) neutralizing antibodies (NAbs) generated during a primary infection are critical for long-term protection against the homologous DENV serotype. However, antibodies that are cross-reactive (CR) but weakly neutralizing can enhance viral replication during a secondary infection with a heterologous serotype via ADE, a phenomenon well documented in both human and animal studies [20,21,22,23]. This paradoxical role of antibodies highlights the need to better understand the qualitative features of the humoral response during primary DENV infections.

One of the most interesting aspects of flavivirus immunity is the dual role of antibodies. Primary DENV infections stimulate type-specific (TS) neutralizing antibodies (NAbs) that are critical for long-term protection against the homologous DENV serotype. From people exposed to sequential DENV infections with different serotypes, investigators have isolated new classes of serotype cross-neutralizing Abs that are also cross-protective in animal models. This complex role of antibodies in DENV pathogenesis highlights the importance of studies that further dissect and better understand humoral immunity to DENVs.

The complexity of the DENV antigenic landscape further complicates our understanding of humoral immunity. The DENV envelope (E) protein includes both TS epitopes unique to each serotype and CR epitopes shared across all four serotypes and with other flaviviruses such as ZIKV [24,25,26,27]. While early studies emphasized epitopes localized to domain III (EDIII) of the E protein, more recent structural and serological analyses have revealed that many highly potent and broadly neutralizing antibodies recognize quaternary structure–dependent epitopes that span adjacent E monomers—such as the E-dimer epitope (EDE)—or target conserved regions within the fusion loop of domain II [28,29,30,31]. These findings underscore that the antigenic surface of DENV is defined not only by discrete domains but also by conformational and inter-dimeric arrangements that collectively shape the breadth and potency of antibody responses.

In the case of the NS1 protein, higher pre-infection anti-NS1 antibody levels have been associated with asymptomatic infection [32], underscoring the potential of NS1-specific immunity in modulating disease outcome.

While the roles of CD8^+^ T cells and neutralizing antibodies in DENV immunity have been extensively studied, the contribution of CD4^+^ T cells—especially follicular helper T cells subset (Tfh)—has received less attention. CD4^+^ T cells are essential for initiating and maintaining germinal center (GC) reactions that support B cell activation, class switching, affinity maturation, and the generation of memory B cells and long-lived plasma cells [33,34,35]. Various mechanisms related to T cell-dependent B cell priming have been characterized extensively in the past. In this current work, we focused on surveying levels of BAFF and IL-21, as both cytokines serve both direct and indirect functions during the process of B cell activation and are produced by CD4^+^ T cells. Both BAFF and IL-21 comprise a select milieu of cytokines produced during co-receptor engagement interactions between activated CD4^+^ T cells and B cells during GC reactions that promote processes such as isotype class switching, antibody-secreting cell differentiation, and memory B cell formation. Because of this, robust antigen-driven activation of CD4^+^ T cells, among other cell types involved in GC reactions, is pivotal for the establishment of such receptor-ligand driven interactions with activated B cells in order to promote higher affinity matured humoral responses [36]. In more detail, engagement of BAFF receptors on B cells such as B-cell maturation antigen (BCMA) receptor has been shown to promote differentiation, plasma cell survival, and plasmablast response activation [37,38,39,40]. In the case of IL-21, this cytokine has been shown to promote GC formation, B cell proliferation, plasma cell differentiation and isotype class-switching, among other functions [41,42]. While we were unable to detect significant differences for those cytokines between the groups, lack of CD4^+^ T cells correlated with an early polyclonal activation of the naïve and IgG positive B cells. That polyclonal activation seems to be potentially less affinity-matured in the depleted group, as it translated into impaired Abs functions including increased non-neutralizing cross-reactivity against DENV1 and 3, limited cross-neutralization against DENV4, and able to amplify ZIKV replication. These mechanisms are critical for shaping the quality, durability, and specificity of the humoral response, particularly in flavivirus infections where CR antibodies may contribute to ADE. However, the mechanistic role of CD4^+^ T cells and its subpopulations in shaping the B cell response during primary DENV infection remain poorly defined [43,44,45]. In particular, it is unknown how CD4^+^ T cell help—or its absence—impacts the emergence of TS versus CR antibodies, the kinetics of class switching, and the potential for cross-reactive but non-neutralizing responses with ADE potential. In this study, we used a non-human primate (NHP) model to interrogate the role of CD4^+^ T cells in a primary DENV2 infection. Rhesus macaques were either depleted of CD4^+^ T cells prior to infection or left undepleted as controls. We assessed the effects of CD4^+^ T cell absence on viral kinetics, B cell activation, antibody specificity, isotype switching, neutralization capacity, and cross-reactivity to other DENV serotypes and ZIKV. To address this gap in knowledge, this study utilizes a non-human primate (NHP) model of CD4^+^ T cell depletion to interrogate their role in shaping protective humoral immunity during a primary DENV2 infection, with direct implications for vaccine development. Our findings reveal that CD4^+^ T cells are pivotal not only for ensuring rapid viral clearance and effective isotype class switching, but also for driving the development of potent, type-specific neutralizing antibodies. Without them, antibody responses were delayed, naïve and memory B cells became aberrantly activated, early neutralization capacity was compromised, and CR antibodies against E and NS1 proteins were amplified—ultimately heightening ZIKV cross-reactivity and the risk of ADE. Together, these insights emphasize how CD4^+^ T cells orchestrate both the quality and longevity of humoral immunity during primary DENV infection and underscore their central importance in designing vaccines that achieve safe and durable protection.

## 2. Materials and Methods

### 2.1. Animal Study Design

A total of 29 young adult male rhesus macaques (*Macaca mulatta*), aged 4–7 years, were housed in the Caribbean Primate Research Center (CPRC) facilities at the University of Puerto Rico, San Juan. Animals were confirmed seronegative for both DENV and ZIKV prior to enrollment in the study using IgM and IgG ELISA assays. All procedures were reviewed and approved by the Institute’s Animal Care and Use Committee (IACUC-UPR-MSC) and performed in a facility accredited by the Association for Assessment and Accreditation of Laboratory Animal Care (AAALAC) (Animal Welfare Assurance number A3421; protocol number, 7890116). In addition, the study was conducted following the USDA Animal Welfare Regulations, the Guide for the Care and Use of Laboratory Animals, and institutional policies, to ameliorate animal suffering by the recommendations of the Weatherall Report on the use of non-human primates in research. Animals were housed with environmental enrichment and all procedures were conducted under anesthesia by intramuscular injection of ketamine at 10–20 mg/kg of body weight, as approved by the IACUC. Anesthesia was delivered in the caudal thigh using a 23-gauge sterile syringe needle. Throughout the study, animals were continuously monitored by trained veterinarians at the Animal Research Center.

Animals were divided into two groups: 12 animals in group 1 (G1) that underwent CD4 T cell depletion, and 17 animals in group 2 (G2) that served as a control group (Figure S1). CD4 T cell depletion was performed using the anti-CD4 monoclonal antibody [clone CD4R1] provided by the Nonhuman Primate Reagent Resource (NHPRR, https://www.nhpreagents.org). This antibody binds to CD4-expressing cells in rhesus macaques. Depletion consisted of an initial subcutaneous (s.c.) administration of 50 mg/kg of anti-CD4 at 15 days pre-infection, followed by two intravenous (i.v.) administrations of 7.5 mg/kg at 13- and 9- days pre-infection. Control animals in G2 were administered phosphate-buffered saline (PBS) in an initial s.c. inoculation of 20 mL at 15 days prior to viral infection, followed by two i.v. injections of 20 mL, 13 days and 9 days before viral infection, respectively.

**Figure 1:**
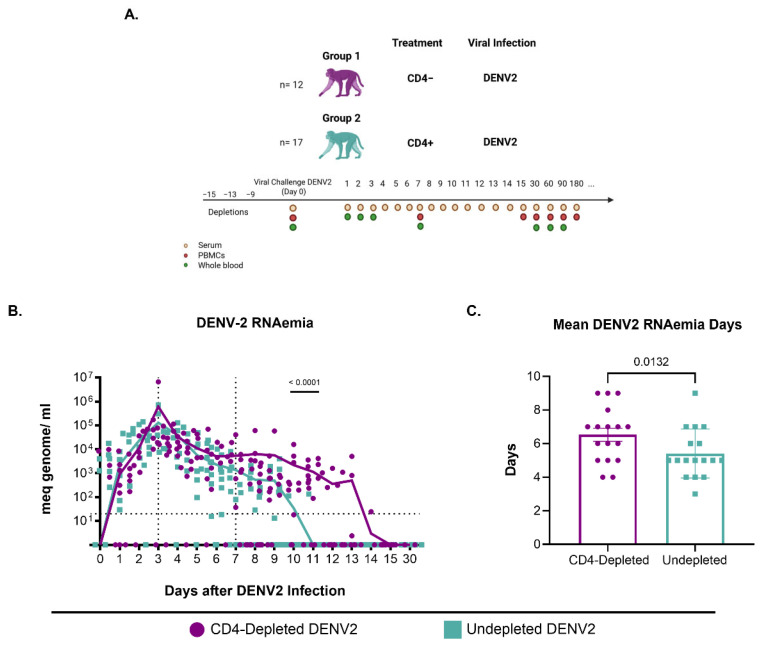
**DENV2 RNA kinetics in flavivirus-naïve CD4-depleted and undepleted animals.** Two cohorts of rhesus macaques (*Macaca mulatta*) underwent depletion of CD4^+^ T cells (or PBS in control group) followed by DENV2 (5 × 10^5^ pfu s.c.) infection at different timepoints. CD4-depleted animals are depicted in purple and undepleted animals are depicted in turquoise. (**A**) Experimental design for sample collection. (**B**) DENV2 genome copies/mL were measured in serum to monitor viral replication during the first 15 days and day 30 after infection. Genome copies per mL are shown logarithmically. DENV RNAemia was defined as early RNAemia (days 0 to 3 p.i.), mid RNAemia (days 4 to 7 p.i.) and late RNAemia (day 7 p.i. onwards). (**C**) Average RNAemia days were calculated using the following formula: total viremia days divided by the total number of days during which viremia was monitored. Statistically significant differences among and within groups were calculated by two-way ANOVA using Tukey’s multiple comparisons test and unpaired multiple *t*-tests followed by Bonferroni’s multiple comparisons test.

Following depletion therapy, animals were infected subcutaneously with 5.0 × 10^5^ plaque-forming units (pfu) of DENV2 NGC strain, denoted from here on as the primary infection (p.i.). The virus stocks were propagated in C6/36 *Aedes albopictus* mosquito cells. Blood samples were collected on days −15, −12, and −9 pre-infection, and during the first fifteen consecutive days after infection, followed by monthly sample collection. Collected samples included serum, heparinized whole blood, and citrate whole blood for PBMC isolation. Macaques were monitored after treatments and infection by trained veterinarians for evidence of disease and clinical status. Rectal and external temperatures and weights were taken on day 0 and every other day pre- and post-infection timepoint. A summary of the experimental design and groups is provided (Figure 1).

### 2.2. Viral Stock

DENV2 New Guinea 44 (NGC) strain (provided by Steve Whitehead, NIH/NIAID, Bethesda, MD, USA) was used to infect macaques at different time points. In addition, DENV2 virus was used for neutralization assays, as well as DENV1 Western Pacific 74, DENV3 Sleman 73, DENV4 Dominique (all three kindly provided by Steve Whitehead (National Institutes of Health, Bethesda, MD, USA), and ZIKV PRVABC59 (ATCC VR-1843) strains. The virus stocks were propagated in C6/36 *Aedes albopictus* mosquito cells. Viruses were expanded and tittered by plaque assay and qRT-PCR using protocols standardized in our laboratories.

### 2.3. Immunophenotyping

Phenotypic characterization of rhesus macaque’s adaptive immune response was performed by 16 -multicolor flow cytometry using fluorochrome-conjugated Abs at several time points. Depletion efficacy was confirmed by measuring the frequency of CD4^+^ T cells on the following timepoints: pre-treatment/depletion baseline (−15), and days −14, −12, and −8 pre-infection. In addition, CD4^+^ T cell frequency was measured on baseline and days 1, 2, 3, 7, 15, 30, 60, and 90 p.i. Aliquots of 150 μL of heparinized whole blood were incubated with a mix of antibodies for 30 min in the dark at room temperature. All heparinized blood samples were processed for flow cytometry within 2 to 4 h after blood draw to ensure high viability; hence, the use of viability dyes was not included in this workflow. After incubation, red blood cells were fixed and lysed with BD FACS fix and lyse solution, and cells were washed twice with BSA 0.05%. Samples were analyzed using a MACSQuant^®^ Analyzer 16 Flow Cytometer (Miltenyi Biotec, San Diego, CA, USA). Antibodies used in this study were: CD8-FITC (DK25) from Sigma; CD3- PerCP (SP34), Ki67- Viogreen (B56), CD4- APC (L200), CD69- PeCy7 (FN50), CD95- PE (DX2), IgG- APC- H7 (G18- 145), CD11b- AF 700 (ICRF44), CD80- BV650 (L307.4), CD27- PE (M-T466) from BD Biosciences (Milpitas, CA, USA); CD20- PacBlue (2H7), HLA-DR- BV570 (L243), CD138- FITC (DL- 101), from Biolegend (San Diego, CA, USA); CD28- APC- Vio770 (I5E8) from Miltenyi (North Rhine-Westphalia, Germany); and CD38- APC (AT-1) from Adipogen (San Diego, CA, USA). For analysis, lymphocytes (LYM) were gated based on their characteristic forward and side scatter patterns. T cells were defined as CD3^+^CD20^-^, CD4^+^ T cells (CD3^+^CD20^-^CD4^+^), CD8^+^ T cells (CD3^+^CD20^-^CD8^+^), and subpopulations were determined within CD4^+^ and CD8^+^ T cells and defined as naïve (CD3^+^CD20^-^CD28^+^CD95^-^), effector memory (EM) (CD3^+^CD20^-^CD28^-^CD95^+^), and central memory (CM) (CD3^+^CD20^-^CD28^+^CD95^+^). Gating strategy for T cells is presented in Supplementary Figure S2. All data from cell population frequencies and immune markers expression were analyzed using Flow/Jo (FlowJo 10.8.1) software and GraphPad Prism (FlowJo LLC., Ashland, OR, USA).

B cells were defined as CD20^+^CD3-, Memory (MBC = CD20^+^, CD3-, CD27^+^), class-switched IgG Memory (IgG MBC = CD20^+^, CD3-, CD27^+^, surface IgG^+^ (sIgG)), and naive/unclass-switched memory B cells (CD3-CD20^+^CD27-). Activated phenotypes were measured via the inclusion of the CD69^+^ marker. Gating strategy for B cells and subsets is presented (Figure S3).

### 2.4. qRT-PCR

DENV2 viral RNA for real-time PCR assay was extracted from 200 μL of virus isolate (previously tittered by FRNT) and from acute serum samples using a MagMax Viral/Pathogen Nucleic Acid Isolation Kit (Applied Biosystems, Waltham, MA, USA) as per the manufacturer’s instructions. Viral RNA extraction was performed using a KingFisher Duo Prime Purification System (Thermo Fisher Scientific, Waltham, MA, USA). In brief, a Viral Pathogen DNA/RNA binding magnetic bead solution is prepared, 200 μL of sera, 500 μL of Viral Pathogen Nucleic Acid isolation kit wash buffer, 5 μL of a protein kinase solution, and 1 mL of ethanol solution (80% ethanol in DNAse free water) were added to specified wells in a KF Duo Deepwell 96 plate (Thermo Fisher Scientific, Waltham, MA, USA) and loaded on the KingFisher Duo Prime Purification System. Purified RNA was collected and stored at −80 °C in kit elution buffer solution. Real-time PCR for DENV2 isolated RNA was carried out using the Genesig Real-Time PCR detection kit for Dengue Virus (Primer Design, Manchester, UK) according to the manufacturer’s protocol (catalog no. oasig-onestep, SKU: R01033). Primers are designed to target the 3′ untranslated region (3′ UTR) of all four DENV serotypes and have 100% homology with over 95% of reference sequences contained in the NCBI database. However, the specific sequences of the primers are proprietary to the manufacturer and are not publicly available, as the company maintains them as confidential to protect their product. Briefly, a kit positive control is diluted in 500 μL of a kit Template buffer and a series of tenfold dilutions were prepared as follows: 2 × 10^5^/μL, 2 × 10^4^/μL, 2 × 10^3^/μL, 2 × 10^2^/μL, 20/μL, 2/μL (copy number/mL). For the reaction mix, 10 uL of oasig 2 X Precision one step qRT-PCR was combined with 1 μL of kit probe/primer mix and 4 μL of sterile water, for a total volume of 20 μL once the RNA sample was added. The reaction mix was added to a 96-well plate, alongside the experimental RNA samples, a kit non-template control (NTC), an RNA extraction negative control (DNAse free water), a positive control (RNA extraction from virus stock), and calibration standards, in specified wells. The plate was then loaded, and the RT-PCR reaction was performed using a Quant Studio 5 Real-Time PCR Instrument (Applied Biosystems, Waltham, MA, USA). For quantification, a standard curve was generated from the ten-fold dilutions of the kit RNA positive control.

### 2.5. ELISAs for Detection of DENV NS1 Antigen and DENV Anti-NS1 IgG

Detection of DENV NS1 antigen and DENV anti-NS1 IgG after primary DENV2 infection was measured using commercial kits (InBios, International Inc., Seattle, MA, USA). The assays were performed as per the manufacturer’s instructions. Timepoints measured include baseline and days 3, 5, 7, 15, and 30 p.i. for DENV NS1, and baseline and days 15, 30, and 90 p.i. for DENV anti-NS1 IgG. For the DENV anti-NS1 IgG assay, no limit of detection or cut-off value is provided because this value will vary depending on flavivirus disease prevalence in the geographical location where the test is performed. For that reason, the cut-off was set to two standard deviations (SD) of the average OD from the readings of negative baseline samples before exposure to the virus.

### 2.6. ELISAs for DENV and ZIKV Antibodies

Serostatus of animals for DENV and ZIKV was assessed before and after DENV2 infection using DENV IgG/IgM commercial kits (Focus Diagnostics, Cypress, CA, USA). ZIKV serostatus was also determined using a commercial kit for ZIKV IgM (InBios, International Inc., Seattle, MA, USA) and ZIKV IgG (XpressBio, Frederick, MD, USA). Timepoints measured include baseline and days 7, 10, 15, 30, 60, and 90 p.i. All assays were performed as per the manufacturer’s instructions and as described by our group [46,47,48]. For the detection of IgG subclasses IgG1 and IgG2, an in-house assay was performed. Briefly, a 96-well plate was coated with DENV-2 antigen at a concentration of 2.5 μg/mL. After coating overnight, plate was washed with PBS T20 and blocked with 5% BSA (Fisher, Waltham, MA, USA) for 1 h at 37 °C. Serum samples were diluted 1:50 in 5% BSA and added to the plate for 1 h 37 °C. Unbound antibodies were removed by washing, followed by the addition of HRP-conjugated anti-rhesus IgG1 or IgG2 secondary Ab (1:3000) (NHPRR, https://www.nhpreagents.org). Lastly, secondary Ab was washed off, and signals were developed with o-phenylenediamine dihydrochloride substrate tablets (Sigma, 34006, St. Louis, MO, USA). Plate wells were read at 492 nm optical density (OD).

### 2.7. Endpoint Dilution Binding Assay

As previously described by our group, an endpoint dilution assay was performed by a capture ELISA assay using samples from a late convalescent timepoint that was 320 days for the CD4-depleted group and 286 days for the undepleted group. For normalization purposes, a reference timepoint of 300 days p.i. was used. Coating buffer (Sigma, 08058) with antigens DENV-1, DENV-2, DENV-3 and DENV-4 (Fitzgerald), or ZIKV (MyBiosource, San Diego, CA, USA) was incubated overnight at 4 °C in a 96 well plate at concentration of 2.5 μg/mL for each antigen. After coating, unbound antigen was washed with PBS 0.05% Tween 20 and further blocked with 5% BSA (Fisher) for 1 h at 37 °C. Serum samples were then serially diluted (1:100, 1:3) using blocking buffer (BSA 5%) and then incubated for 1 h at 37 °C. All unbound antibodies were removed by washing, followed by incubation for 1 h at 37 °C with goat-anti-human secondary Ab conjugated with horseradish peroxidase (HRP) (Bio-Rad, Hercules, CA, USA). Lastly, unbound secondary Ab was washed off, and signals were developed with o-phenylenediamine dihydrochloride substrate tablets (Sigma, 34006). Plate wells were read at 492 nm optical density (OD).

### 2.8. EDIII and NS1 Antigen Production

ZIKV (H/PF/2013) EDIII antigen used in this study was produced as previously described [49]. EDIIIs of all four DENV serotypes were also cloned and expressed similarly to ZIKV EDIII. Briefly, codon-optimized genes encoding the EDIIIs of DENV1 (AAB70694·1, E protein aa 297-394), DENV2 (ADA00411·1, E protein aa 297-394), DENV3 (AAB69126·2, E protein aa 295-392) and DENV4 (ADA00410·1, E protein aa 297-394) with an N-terminal human serum albumin signal peptide, a polyhistidine tag, and a HaloTag were cloned into the mammalian expression plasmid pαH. Recombinant DENV EDIII antigens were expressed in mammalian Expi293 cells and purified from the culture supernatant using Ni-NTA agarose (Qiagen, Venlo, Limburg, The Netherlands). Purified EDIII antigens were biotinylated using HaloTag PEG biotin ligand (Promega, Madison, WI, USA), according to the manufacturer’s protocol. For NS1 antigen production, codon-optimized genes encoding the NS1s of DENV1 NS1 Region 37, DENV2 NS1 Region 39, DENV3 NS1 Region 22, DENV4 NS1 Region 45 and ZIKV NS1 Region 47 were used. Mobility-shift analysis was used to assess the biotinylated EDIII antigens using SDS-PAGE. The mammalian expression plasmids and sequences will be made available in the plasmid repository and Genbank, respectively.

### 2.9. Coupling of EDIII Antigens to Beads via Avidin-Biotin Interaction

Site-specifically biotinylated EDIII antigens were coupled to unique MagPlex^®^-Avidin Microspheres. Antigen reading regions (specific antigen location in the microsphere bead) for the Luminex signal detection were, BSA (region 22), DENV1 (region 12), DENV2 (region 26), DENV3 (region 36), DENV4 (region 38), and ZIKV (region 30) at a concentration of 5 μg of antigen per 106 beads in phosphate-buffered saline, for 1 h at 37 °C with shaking at 700 rpm. Beads were then washed/blocked three times in blocking buffer (PBS 1% BSA) and resuspended in wash buffer at 2 × 10^6^ beads/mL.

### 2.10. Coupling of Biotinylated NS1 Antigens to Beads via Avidin-His-Tag Ab Interaction

Site-specifically biotinylated NS1 antigens were coupled to unique MagPlex^®^-Avidin Microspheres using a His-Tag antibody incubated for 1 h at 37 °C with shaking at 700 rpm, prior to the NS1 antigen coupling. Antigen reading regions (specific antigen location in the microsphere bead) for the Luminex signal detection were, BSA (region 22), DENV1 (region 37), DENV2 (region 39), DENV3 (region 14), DENV4 (region 45), and ZIKV (region 47). Following the His-tag Ab coupling microspheres, specific biotinylated NS1 antigen was added at a concentration of 5 μg of antigen per 106 beads in PBS for 1 h at 37 °C shaking at 700 rpm. Beads were then washed/blocked three times in blocking buffer (PBS + 1% BSA) and resuspended in wash buffer at 2 × 10^6^ beads/mL.

### 2.11. EDIII and NS1 Multiplex Assay

DENV and ZIKV EDIII coupled beads were combined in equal ratios by plating 50 μL containing 2500 beads per antigen into each well of a 96-well assay plate (Cat. No:655906). Rhesus macaque serum samples from 90 days p.i. were diluted 1:500 in blocking buffer. Mouse monoclonal antibodies specific for each EDIII antigen ZV-67 (ZIKV), E103 (DENV1), 3H5 (DENV2, 8A1 (DENV3), E88 (DENV4) and NS1 antigen were titrated in each assay as controls for assay consistency across couplings, plates, and assay days. A magnetic plate separator was used to remove the blocking buffer from the assay plate, and 50 μL of diluted serum or mAb was added to the beads. The serum and bead mixture were incubated for 1 h at 37 °C shaking at 700 rpm. Beads were washed twice with 200 μL blocking buffer per well using a magnetic separator. Following blocking, 50 μL of Goat anti-Human IgG Fc Multi-species SP ads-PE (Southern Biotech, catalog:2014-09) and Goat anti-Mouse IgG Fc Human ads-PE (Southern Biotech, catalog:1030-09) were added to the beads (6 μg/mL in block buffer) and incubated for 1 h at 37 °C shaking at 700 rpm to detect respective antibody species. Beads were washed thrice with 200 μL blocking buffer per well using a magnetic separator and resuspended in 100 μL of blocking buffer before acquiring data. Fluorescence data was obtained using a Luminex 200 analyzer set to acquire at least 50 beads per bead region.

### 2.12. In Vitro ADE Assay in K562 Cells

The ability of serum antibodies from DENV2-immune animals to enhance ZIKV was assessed in K562 cells (ATCC CCL-243). Briefly, macaque serum from 90 days p.i. was diluted three-fold starting after an initial dilution of 1:25 and then incubated with the virus of interest at an approximate multiplicity of infection (MOI) of 1.0 for 1 h at 37 °C. Monoclonal antibody 4G2 was used as a positive control at 0.5 mg/mL. Approximately 1 × 10^6^ cells were added to each well in a 96-well plate containing the mixture of virus and serum. After incubation at 37 °C for 2 h, cells were washed with fresh media twice and incubated at 37 °C for 48 h. After incubation, cells were washed, fixed with 1% PFA, permeabilized with saponin and stained with a rabbit monoclonal 4G2 antibody at 4 μg/mL. After incubating 1 h at 4 °C, cells were washed and stained with a secondary goat anti-rabbit IgG conjugated to AlexaFluor-647 at 1:5000 for 1 h at 4 °C. After washing, cells were resuspended in 200 μL of PBS until ready to run on flow cytometer. Percent of infection was determined using an Attune N × T flow cytometer.

### 2.13. DENV Neutralization Assays

Focus Reduction Neutralization Test (FRNT) for all four DENV serotypes (*DENV1 Western Pacific 74, DENV2 New Guinea 44 (NGC), DENV3 Sleman 73, and DENV4 Dominique*) were completed for baseline and days 15, 30, 90 and 300 p.i. Data on neutralization curves for baseline was not included as all animals showed no neutralizing potential and remained below threshold (below 60% reduction). The exact late convalescent timepoint was 320 days for the CD4-depleted group and 286 days for the undepleted group, but for normalization purposes, a reference timepoint of 300 days p.i. was used. Vero81 cells (ATCC CCL-81) were seeded at approx. 2.0 × 10^5^ per well in 96-well plates with DMEM (Dulbecco’s Modified Eagle’s medium, Thermo Fisher Scientific, Waltham, MA, USA) for approx. 18 h. Serum dilutions (ten-fold) were prepared in diluent medium Opti-MEM (Invitrogen) with 2% FBS (Gibco) and 1% antibiotic/antimycotic (Hyclone). Virus was added to each dilution and incubated for 1 h at 37 °C/5%. Before inoculation, the growth medium was removed, and cells were inoculated with 50 μL per well of serum-virus dilution in triplicates; plates were incubated for 1 h at 37 °C/5%CO_2_/rocking. After incubation, 125 μL per well of overlay (Opti-MEM 1% carboxymethylcellulose (Sigma), 2% FBS, 1% non-essential amino acids (Gibco), and 1% antibiotic/antimycotic (HyClone) was added to the plates containing virus dilutions, followed by an incubation period of 45–48 h at 37 °C/5%CO_2_. After two days, overlay was removed with PBS and fixed with 4% paraformaldehyde for 30 min. Plates were blocked with 5% non-fat dairy milk in 1× perm buffer (BD Cytofix/Cytoperm) for 10 min. and incubated for 1 h/37 °C/5%CO_2_/rocking with anti-E mAb 4G2 and anti-prM mAb 2H2 (kindly provided by Dr. Aravinda de Silva and Ralph Baric, University of North Carolina Chapel Hill, Chapel Hill, NC, USA), both diluted 1:100 in blocking buffer. Plates were washed three times with PBS and incubated 1 h/37 °C/5%CO_2_/rocking with horseradish peroxidase (HRP)-conjugated goat anti-mouse antibody (Sigma, Kanagawa, Japan), diluted 1:1500 in blocking buffer. Foci were developed with TrueBlue HRP substrate (KPL) and counted using an Elispot reader. In brief, we establish titers as the FRNT endpoint dilution showing 60% or greater reduction in DENV foci (FRNT60). In addition, we calculated the 50% neutralization (EC50).

### 2.14. ZIKV Neutralization Assays

For the Plaque Reduction Neutralization Test (PRNT) for ZIKV, Vero81 cells (ATCC CCL-81) were seeded at approx. 2.0 × 10^5^ per well in 24-well plates with DMEM (Dulbecco’s Modified Eagle’s medium, Thermo Fisher Scientific, Waltham, MA, USA) for approx. 18 h. Serum dilutions (ten-fold) were prepared in a diluent medium (Opti-MEM (Invitrogen, Waltham, MA, USA) with 2% FBS (Gibco, Waltham, MA, USA) and 1% antibiotic/antimycotic (Hyclone, Logan, Ut, USA). Virus was added to each dilution and incubated for 1 h at 37 °C/5%/CO_2_. Before inoculation, the growth medium was removed, and cells were inoculated with 100 μL per well of each dilution in triplicates; plates were incubated for 1 h at 37 °C/5%CO_2_/rocking. After incubation, 1 mL per well of overlay (Opti-MEM 1% carboxymethylcellulose (Sigma, Kanagawa, Japan), 2% FBS, 1% non-essential amino acids (Gibco, Waltham, MA, USA), and 1% antibiotic/antimycotic (HyClone, Logan, UT, USA) was added to the plates containing virus dilutions, followed by an incubation period of 4 days at 37 °C/5% CO_2_. After four days of incubation at 37 °C/5%CO_2_, the overlay was removed; the cells were washed twice with phosphate-buffered saline (PBS), fixed in 80% methanol, and stained with crystal violet and foci were counted. The mean focus diameter was calculated from approx. twenty foci per clone were measured at 35 magnifications. Results were reported as the PRNT with a 60% or greater reduction in ZIKV or DENV plaques (PRNT60).

### 2.15. BAFF ELISA

Determination of B-cell activating factor (BAFF) levels in sera was assessed using the Monkey BAFF ELISA commercial kit (MyBioSource, San Diego, CA, USA). This Quantitative Sandwich ELISA kit uses coated anti-BAFF antibodies and a BAFF HRP-conjugate reagent technique for analyte detection. The assay was performed per the manufacturer’s instructions.

### 2.16. Serum Cytokines Assay

Detection of serum cytokines after primary DENV2 infection was measured using NHP XL Cytokine Luminex Performance Assay Kit (Bio-techne, R&D systems, Minneapolis, MN, USA). The assays were performed per the manufacturer’s instructions. First, 50 μL of standard, control, or sample were mixed with 50 μL of diluted microparticle cocktail and added to each well. The mixture was incubated for 2 h at room temperature on a shaker at 800 rpm. Three wash steps using 100 μL of wash buffer were performed. Then, 50 μL of diluted biotin-antibody cocktail was added to each well, covered, and incubated for 1 h at room temperature on a shaker at 800 rpm. The last step of washing was performed followed by a resuspension of the beads before reading within 90 min using a Luminex analyzer. Cytokines surveyed in this assay include: IFN alpha, BCA-1, IP-10, IL-17A, IL-28A, IL-12 (p70), IL-4, IL-21, IFN-Gamma, MIP-3 alpha, sCD40L.

### 2.17. Quantification and Statistical Analysis

Statistical analyses were performed using GraphPad Prism 9.0 software (GraphPad Software, San Diego, CA, USA). For viral burden analysis, the log titers and levels of vRNA were analyzed via multiple unpaired *t*-tests and two-way ANOVA. The statistical significance between or within groups evaluated at different time points was determined using one-way or two-way analysis of variance (ANOVA) (Tukey’s, Sidak’s, or Dunnett’s multiple comparisons test) or unpaired *t*-test to compare the means. Significant multiplicity-adjusted *p*-values (* < 0.05, ** < 0.01, *** < 0.001, **** < 0.0001) show statistically significant differences between groups (Tukey test) or time points within a group (Dunnett test).

## 3. Results

### 3.1. CD4^+^ T Cell Depletion Impairs Viral Clearance Following Primary DENV2 Infection

To evaluate CD4^+^ T cell depletion effectiveness, we assessed CD4^+^ T cell frequencies in fresh heparinized blood using immunophenotyping via flow cytometry at pre-treatment (pre-TX), post-treatment (post-TX), and after primary DENV2 infection. Prior to treatment, CD4^+^ T cell frequencies were similar between groups (mean = 61.9 ± 0 for G1 and 63.3 ± 0 for G2). By day 2 post-TX, CD4^+^ T cells were reduced by 99.8% in G1 (Figure S1A). This reduction was consistent until approximately day 7 p.i., when they began to gradually recover. Of note, by day 300 p.i. CD4^+^ T cell frequency had not returned to baseline levels in depleted animals, demonstrating that a complete recovery does not occur in comparison to control animals.

Interestingly, the absence of CD4^+^ T cells in depleted animals promoted an increase in CD8^+^ T cell frequency (Figure S1B). To determine if CD8^+^ T cell integrity was affected, we assessed CD8^+^ expression of central and effector memories markers and observed no differences between groups (Figure S1C,D). This suggests that CD4^+^ T cell depletion did not promote dysfunctional surface expression markers on CD8^+^ T cell response. The gating strategy used for T cell immunophenotyping can be found in Supplementary Figure S2.

We found that CD4^+^ T cell correlates with enhanced DENV2 replication assessed by two different methods, RNAemia or NS1 protein expression. DENV2 RNAemia was measured in serum during the first 15 days and day 30 p.i. (Figure 1A). We defined RNAemia as early (days 1 to 3 p.i.), mid (days 4 to 7 p.i.), and late (day 8 p.i. onwards). During the early period, viral RNA detection increased similarly in all groups (Figure 1B). However, by day 5 p.i., the control group began to show a consistent decline in viremia, leading to resolution by day 10 p.i., with only one animal showing viremia on day 11 p.i. In contrast, the CD4-depleted group maintained higher detectable DENV2 RNAemia levels after day 10 p.i. This difference was statistically significant on days 10 and 11 p.i. (*p* = 0.000078 and *p* = 0.001346, respectively). By day 12 p.i., there was no viral RNA detection in any animal from the control group, and, by day 15 p.i., all animals in both groups tested negative for DENV2 (Figure 1B and Table 1). These results indicate a faster RNAemia resolution in the CD4^+^ T-cell-competent group compared to CD4-depleted animals and a delay in viral clearance in animals that are CD4-deficient. Next, we evaluated the average RNAemia days, defined as the number of days with detectable viremia divided by the number of animals per group (Figure 1C). The undepleted animals had significantly fewer mean RNAemia days compared to the depleted animals (*p* = 0.0132), suggesting that CD4^+^ T cells have a role in controlling DENV2 RNAemia, but whether this effect is mediated directly by CD4 T cells, by an interaction with B cells or a combination of both activities, remains to be determined. To independently confirm viremia and to explore the role of CD4^+^ T cells in NS1 antigenemia, we measured DENV NS1 levels in serum. NS1 has been shown to correlate with virus replication and the level of viremia in infected individuals [50,51]. In CD4-depleted and control animals we measured NS1 levels in serum at days 3, 5, 7, 10, 15, and 30 post-DENV2 infection (Figure S4). We observed a similar increase in serum NS1 levels over the first 7 days p.i. Between days 10 and 15 p.i. NS1 levels in the control group declined, whereas levels in the CD4-depleted group continued to increase and peaked by day 15 p.i. This suggests that the lack of CD4^+^ T cells enhances DENV2 replication and NS1 levels in circulation, further confirming a role for CD4^+^ T cells in viral clearance during a primary infection.

Lastly, to determine any effects of DENV viral infection on NHPs, a range of clinical parameters and complete blood counts were monitored before and after DENV2 infection (Figure S5). No significant differences were observed between groups.

### 3.2. Lack of CD4^+^ T Cells Constrains Anti-DENV2 Antibody Induction

To assess the impact of CD4^+^ T cell depletion on Ab responses to primary DENV2 infection, we measured virus-specific IgM and IgG levels after infection (Figure S6). The induction of virus-specific IgM was delayed and lower in magnitude in CD4-depleted animals compared to control animals at days 10 and 15 p.i. (*p* < 0.0001) (Figure S6A). Furthermore, total anti-DENV IgM produced from day 0 to day 15 p.i. was also significantly lower in the CD4-depleted group in comparison to the control group (*p* = 0.0007) (Figure S6B). While anti-DENV IgG levels were statistically different between both groups only on day 15 p.i., where it appears that the CD4-depleted group was slower, by ~45 days to generate as robust an IgG response compared to the control group (*p* < 0.0001) (Figure S6C). Similar results were observed with the total anti-DENV IgG produced (from day 0 to day 60), where CD4-depleted animals had significantly lower IgG values compared to undepleted animals (*p* = 0.0153) (Figure S6D). Virus-specific IgG levels were comparable in both groups at convalescence (days 60 and 90 p.i.). Taken together, these results demonstrate that CD4^+^ T cell depletion causes a delay in activated B cells ability to produce antigen specific antibodies during primary DENV2 infection. To further assess the impact of CD4^+^ T cell depletions on the Ab response, we measured IgG subclasses IgG1 and IgG2 after infection (Figure S7). An increase in IgG1 levels was observed through days 15, 30, 90, and 300 p.i., compared to baseline in both groups. However, at day 15 p.i., a significant difference was observed, where the undepleted group showed higher levels of IgG1 compared to the depleted group (*p* < 0.0001). By day 30 p.i., both groups showed similar levels of IgG1. Interestingly, by days 90 and 300 p.i., a shift is observed where the depleted group showed significantly higher levels of IgG1 compared to the undepleted group (*p* < 0.0001). On the other hand, IgG2 was not detected. Taken together, these results show that a lack of CD4^+^ T cell priming can lead to a delay in IgG1 production. Furthermore, the Ab response elicited by a primary DENV2 infection does not promote a robust selection of IgG2 Ab production.

### 3.3. DENV Anti-NS1 Abs Are Modified by a Lack of CD4^+^ T Cells

Next, we compared anti-DENV NS1 IgG levels in both groups at days 15, 30, and 90 p.i. (Figure S8). No DENV NS1 anti-IgG antibodies were detected on day 0 as expected. By day 15 p.i., CD4-depleted animals had significantly lower levels in comparison to the undepleted animals, similar to anti-DENV IgG levels previously presented (*p* = 0.000208). By day 30 p.i., both groups had reached similar anti-NS1 IgG levels. Interestingly, by day 90 p.i., CD4-depleted animals showed significantly higher anti-NS1 IgG levels compared to undepleted animals (*p* = 0.000208) and, surprisingly, the Abs were still detected only in that group one year after DENV2 infection (*p* < 0.0001). These results demonstrate an initial delay in the development of NS1 binding IgG in the CD4-depleted group followed by an increase NS1Ab levels at late convalescence compared to the control group.

Altogether, this result supports an initial delay in Ab switch in this group, as seen in the total IgG Abs response followed by an expansion of anti-NS1 antibodies by day 90 p.i., which is sustained at least one year p.i. but only in the absence of CD4^+^ T cells. This fact strongly suggests the development of a population of cross-reacting non-specific anti-NS1 Abs in the late convalescent period associated with a blunted priming of B cells.

### 3.4. Depletion of CD4^+^ T Cells Modifies IgG-Binding Capacities of Antibodies Against Heterologous Dengue After Primary DENV2 Infection

**Figure 2:**
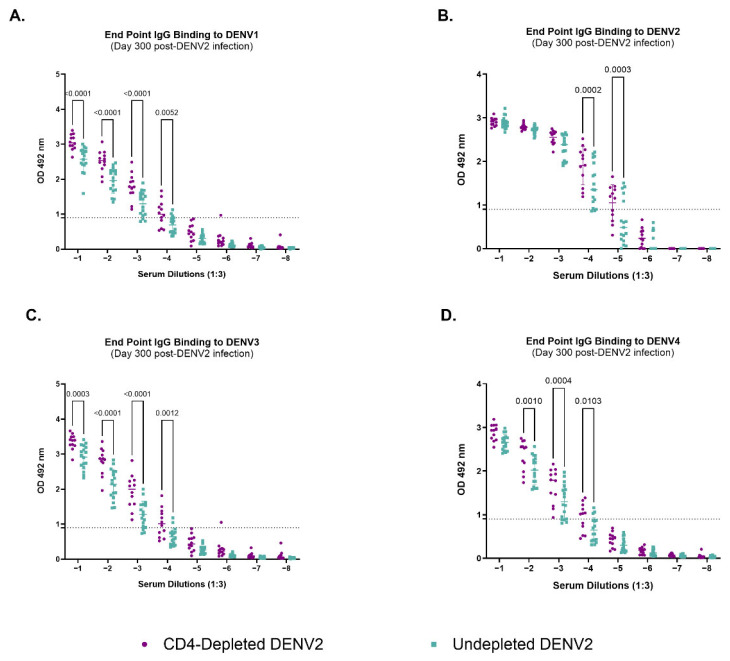
**Depletion of CD4^+^ T cells modifies the binding capacity of antibodies against DENV.** The quality of the IgG humoral immune response was accessed using an endpoint dilution binding Elisa. Serum of 300 days post primary DENV2 infection was used. CD4-depleted animals are depicted in purple and undepleted animals are depicted in turquoise. (**A**–**D**) IgG binding results against all DENV serotypes are shown from 300 days post-DENV2 infection. Dotted lines represent cut off value. Statistical analysis among groups were observed using two-way ANOVA to compare the values of CD4-depleted DENV2 and undepleted groups.

To further explore the role of CD4^+^ T cells, we assessed their impact on the quantity and quality of the humoral response against all four DENV serotypes and ZIKV *(DENV serotypes: (DENV1 Western Pacific 74, DENV2 New Guinea 44 (NGC), DENV3 Sleman 73, DENV4 Dominique) and ZIKV (PRVABC59)* 300 days after DENV2 infection using the endpoint dilution approach (Figure 2 and Figure S9). No binding activity against ZIKV was observed (Figure S9). On the other hand, the CD4-depleted group showed significantly higher levels of IgG binding capabilities across serum dilutions against DENV1 (Figure 2A) (*p* < 0.0001, *p* < 0.0001, *p* < 0.0001 and *p* = 0.0052) DENV2 (Figure 2B) (*p* = 0002 and *p* = 0.0003), DENV3 (Figure 2C) (*p* = 0.0003, *p*< 0.0001, *p* < 0.0001 and *p* = 0.0012) and DENV4 (Figure 2D) (*p* = 0.0010, *p* = 0.0004 and 0.0103) when compared to the undepleted animals. However, these antibodies seem to be of high-neutralizing capacity only against DENV2 and DENV4, as seen in our neutralization data on days 90 and 300 p.i. Binding activity was similar at lower serum dilutions between the control and depleted group for DENV2 (10^−1^–10^−3^) and for DENV4 (10^−1^). However, binding activity against DENV1 and 3 was significantly higher in the depleted group at all serum dilutions. This noteworthy finding strongly suggests that CD4^+^ T cells differentially impact the antibodies repertoire, at functional level, against the autologous vs. heterologous serotypes. Together, these results suggest a significant role of CD4^+^ T cells shaping humoral immune responses, particularly the antibody generation during a primary DENV2 infection.

### 3.5. Lack of CD4^+^ T Cells During a Primary DENV2 Infection Reshapes Abs Binding to the Envelope EDIII Domain and to the NS1 Protein

**Figure 3:**
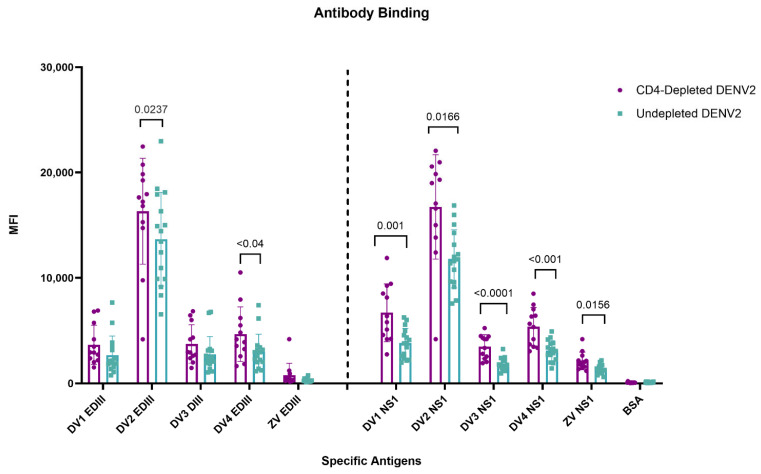
**Epitope reactivity against DENV and ZIKV antigens in depleted and undepleted macaques after DENV2 infection.** Antibody binding against EDIII and NS1 antigens were assessed by Luminex using samples from 90 days after DENV2 infection. CD4-depleted animals are depicted in purple and undepleted animals are depicted in turquoise. Antibody response was measured to EDIII (**left side**) and NS1 (**right side**) antigens from all four DENV serotypes and ZIKV. Significant differences among groups were calculated via two-way ANOVA using the Tukey’s multiple comparisons test and significant differences between groups were calculated using multiple *T* tests.

To further explore the role of CD4^+^ T cells in shaping the antibody response against DENV and cross-reactivity to ZIKV, domain specificity against EDIII and NS1 was quantified using a Luminex assay. The envelope (ENV) EDIII domain was selected as a major neutralizing target and NS1 due to its proposed role in pathogenesis [52,53]. Type-specific and cross-reactive responses were measured at day 90 p.i. after DENV2 infection against EDIII and NS1 antigens from all four DENV serotypes and ZIKV (Figure 3). When analyzing mean fluorescence intensity (MFI) after the primary DENV2 infection, both groups of animals had significantly higher responses against EDIII to the infecting serotype DENV2 compared to the other DENV serotypes and ZIKV EDIII. However, the depleted group showed significantly higher MFI values against DENV2 (*p* = 0.0237) and an extended breadth of binding to DENV4 EDIII region (*p* < 0.04), while a limited cross-reactivity against EDIII to the other DENV serotypes was observed, without differences between groups, and virtually no reactivity to ZIKV EDIII (Figure 3). In contrast to the response to the EDIII domain, a greater breadth of NS1 Ab repertoire was present in connection with the lack of CD4^+^ T cells. Antibodies against DENV2 NS1 were significantly higher in both the depleted and undepleted groups compared to the NS1 from the other DENV serotypes. However, MFI values were significantly higher against all DENV NS1 serotypes in the depleted group (DENV1, *p* < 0.0011; DENV2, *p* < 0.0166; DENV3, *p* < 0.0003; DENV4, *p* < 0.0022). Interestingly, there was a limited but detectable cross-reactivity against the ZIKV NS1 protein in both groups with a significantly higher cross-reactivity in the CD4-depleted group (*p* < 0.0156) (Figure 3). This suggests that during a primary DENV infection, CD4^+^ T cells have a different weight/effect in guiding the Ab responses against the EDIII domain and NS1 protein, potentially through differential support of Tfh populations or through differential recognition of the envelope protein versus NS1 by CD4 T cells [54].

### 3.6. Cross-Reactivity Is Increased by Lack of CD4 T Cells

To confirm the Luminex assay results that depletion of CD4^+^ T cells altered the relative levels of DENV2 TS and flavivirus CR Abs, we measured anti-NS1 ZIKV IgG levels with a different assay and at different timepoints after infection. As shown in (Figure S10), anti-ZIKV IgG develops in the convalescent phase at day 30 p.i. and continues to increase in both groups by days 60 and 90 p.i. and were still detectable one year (365 days p.i) later. Interestingly, the levels of those CR Abs were significantly higher on days 90 and 365 p.i. in the group lacking CD4^+^ T cells (*p* < 0.0001). This result, together with the ZIKV end point binding result (Figure S9), suggests an increased expansion of flavivirus-CR B cell clones in animals without CD4^+^ T cells during the primary DENV infection.

### 3.7. Absence of CD4^+^ T Cells Modifies Antibody Repertoire Properties Increasing ZIKV Replication

**Figure 4:**
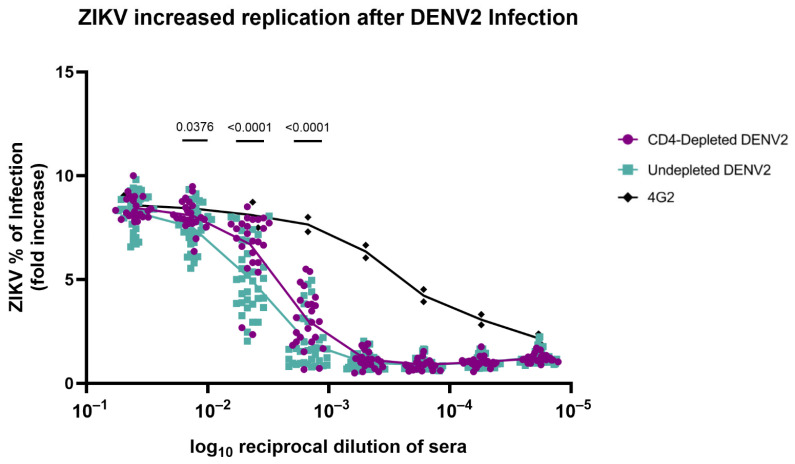
**Depletion of CD4^+^ T cells enhances antibody properties to increase ZIKV replication.** In vitro ADE assay of ZIKV using sera 90 days after DENV2 infection from CD4-depleted and undepleted animals is shown. CD4-depleted animals are depicted in purple and undepleted animals are depicted in turquoise. Significant differences among groups were calculated by two-way ANOVA using the Sidak’s multiple comparisons test.

An in vitro ADE assay with K562 cells was performed to measure ZIKV infection-enhancing antibodies in DENV2-immune macaque serum from 90 days p.i. Sera from both CD4-depleted and undepleted animals exhibited similar ZIKV infection activity at the lowest serum dilution, ranging from 40 to 50% (Figure 4). However, ZIKV replication significantly increased across higher serum dilutions (particularly 1:100, 1:1000, and 1:10,000) in CD4-depleted animals in comparison with the undepleted control group (*p* = 0.0376, *p* < 0.0001 and *p* < 0.0001, respectively). This denotes that a lack of CD4^+^ T cells has an effect on the Ab repertoire that leads to an increase in ZIKV infection compared to undepleted animals and to previously reported naïve animals [46]. These findings highlight the importance of proper CD4^+^ T cell priming in shaping not only the magnitude but also the quality of antibody responses, with important implications for vaccine safety, particularly in flavivirus-endemic regions.

### 3.8. Dynamic of Specific DENV2 and Heterologous DENV4 Neutralizations Are Modulated by a Lack of CD4^+^ T Cells

**Figure 5:**
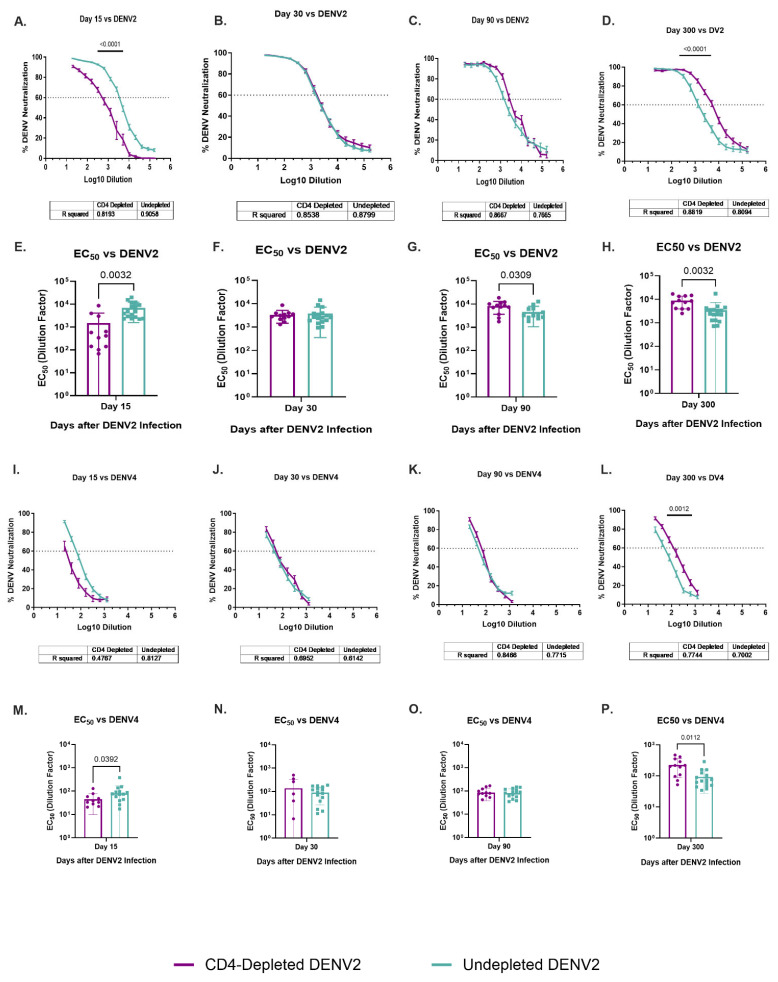
**FRNT values and geometric mean titers of DENV2 and DENV4 neutralizing antibodies.** (**A**–**P**) FRNT60 and EC50 values of neutralizing antibodies against DENV2 and DENV4 after DENV2 infection are shown. CD4-depleted animals are depicted in purple and undepleted animals are depicted in turquoise. Statistically significant differences among groups were calculated by one-way and two-way ANOVA using the Tukey’s multiple comparisons test and unpaired *t*-tests.

To determine the contribution of CD4^+^ T cells to the development and maintenance of the neutralization antibodies (NAbs) response, all animals were tested using plaque reduction neutralization tests (PRNT) and focus reduction neutralization test (FRNT) assays against ZIKV and all DENV serotypes, respectively (Figure 5 and Figure S11). Neutralization assays were completed for baseline and days 15, 30, 90 and 300 p.i. As expected, no animals had NAbs against any flavivirus at baseline. No trends or significant differences were identified in the cross-neutralization at any tested time point against the DENV1 and DENV3 serotypes (Figure S11), and no neutralization of ZIKV was observed at any timepoint (Figure S11).

However, both groups developed neutralizing activity against the infecting serotype DENV2 and the heterologous serotype 4 (Figure 5). Undepleted animals had significantly higher neutralization levels against DENV2 by day 15 p.i. (*p* < 0.0001) in comparison to the depleted animals (Figure 5A). On later timepoints, neutralization was similar between groups. However, by day 300 p.i., a shift is observed where CD4-depleted animals have significantly higher neutralization levels than the undepleted animals (*p* < 0.0001) (Figure 5D). A similar effect is observed in the neutralization against DENV4, where undepleted animals have higher levels on day 15 p.i. (Figure 5I) (although not reaching statistical significance) but, by day 300 p.i., CD4-depleted animals have significantly higher levels than undepleted animals (*p* = 0.0012) (Figure 5L).

The 50% effective concentration (EC50) of neutralizing Abs is also shown. By day 15 p.i., a significantly lower neutralizing activity is observed in CD4-depleted animals in comparison to the undepleted group, not only against DENV2 (*p* = 0.0032) but against DENV4 (*p* = 0.0392) too (Figure 5E,M). This finding is interesting because DENV4 was the only heterologous serotype showing cross-reactivity against EDIII domain in previously discussed results, suggesting that Abs against DENV4 EDIII are indeed cross-reactive but non-neutralizing at early time points and the response evolves to include antibody neutralization. However, CD4-depletion was associated with a significantly higher EC50 against DENV2 compared to the undepleted group at day 90 p.i. (*p* = 0.0309) and against both DENV2 and DENV4 after one year of DENV infection (*p* = 0.0032 and *p* = 0.0112, respectively) (Figure 5G,H,P). These data are of great interest as they suggest a possible delayed neutralizing response against homologous DENV2 in animals lacking DENV2-primed CD4^+^ T cells, which was extended to a heterologous serotype (DENV4).

### 3.9. Lack of CD4^+^ T Cell Priming Promotes Increased IgG^+^ MBC Polyclonal Activation During a Primary DENV2 Infection

To determine the contribution of CD4^+^ T cells in modulating the activation of an naïve/unclass-switched memory B cell (defined as IgM^+^ B cells CD3-CD20^+^CD27-), response to a primary DENV2 infection, whole blood for immunostaining was collected on days 0 (baseline), 7, 15, and 30 p.i. with DENV2, where cell population levels were measured via flow cytometry. The gating strategy is shown in Supplementary Figure S3 While we did not observe significant quantitative differences in naïve/unclass-switched memory B cells between the CD4-depleted and undepleted groups in any timepoint post-infection, a trend is observed where the undepleted group shows higher levels of naïve/unclass-switched memory B cell subset, compared to the CD4-depleted cohort (Figure S12A). Another B cell subtype we assessed was activated naïve/unclass-switched memory B cells (IgM^+^-MBC, defined as CD3-CD20^+^CD27-CD69^+^) (Figure S12B). Here, we observed that the CD4-depleted group showed a significant early expansion of the activated naïve/unclass-switched memory B cell frequency on day 7 p.i. compared to baseline (*p* = 0.00332). However, both groups showed an increase in frequency compared to baseline in the subsequent measured time points. Taken together, this data suggests that activation pathways of naïve/unclass-switched memory B cell clones during a primary DENV2 infection can follow both T cell-dependent or T cell-independent pathways.

**Figure 6:**
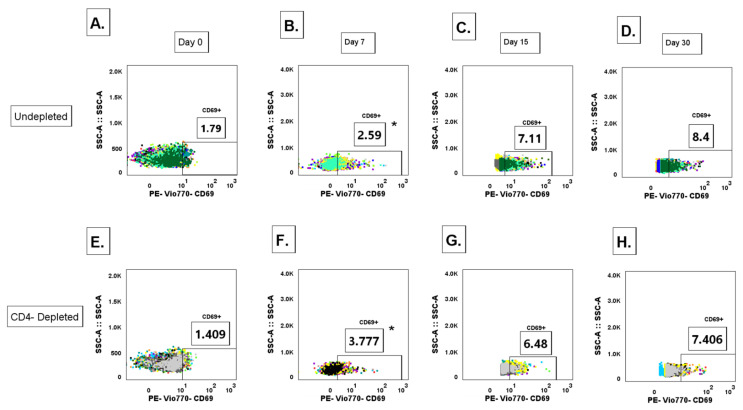
**CD4^+^ T cell depletion promotes polyclonal IgG memory B cell formation activation during DENV2 infection.** Frequencies of activated IgG^+^ memory B cells in CD4^+^ T cell competent and -deficient animals during DENV2 infection. Activated IgG^+^ MBCs frequencies were surveyed via Flow Cytometry during days 0 (baseline), 7, 15, and 30 p.i. during a primary DENV2 infection. This cell population was defined as: CD3-CD20^+^CD27^+^sIgG^+^CD69^+^ where the final gating strategy is shown as percentages of CD69^+^ cells in total IgG^+^ MBCs (CD3-CD20^+^CD27^+^sIgG^+^). (**A**–**D**) Proportions as percentages of parent gate are indicated in encased values shown in the Undepleted control group. (**E**–**H**) Proportions as percentages of parent gate are indicated in encased values shown in the CD4 Depleted group. Population color scheme is representative of each individual animal belonging to its respective group and timepoint. Statistical differences among groups were calculated by two-way ANOVA using Šidák’s and Turkey’s multiple comparisons test and paired multiple *t*-tests. Significant multiplicity-adjusted *p*-values: *p* < 0.05, shown as *.

To determine the contribution of CD4^+^ T cells in modulating the formation and activation of IgG^+^ memory B cell (IgG^+^ MBC) (CD3-CD20^+^CD27^+^sIgG^+^) populations during a primary DENV2 infection, we measured expression levels of surface IgG in total MBCs via flow cytometry. Total IgG^+^ MBC levels were surveyed, and no significant differences were observed in any of the time points measured nor between both groups post-infection (Figure S12C). However, when measuring activated IgG^+^ MBCs (CD3-CD20^+^CD27^+^sIgG^+^CD69^+^), we observed a significant early expansion of these cell types in the CD4-depleted group at day 7 p.i. compared to baseline levels (*p* = 0.0450) (Figure 6 and Figure S12D). Interestingly, on day 7 p.i. the CD4-depleted group showed significantly higher levels of activated IgG^+^ MBCs compared to the undepleted control (*p* = 0.0454). This expansion in the CD4-depleted cohort continued to significantly increase on day 15 p.i., compared to day 7 p.i. (*p* = 0.0100). In the case of the undepleted control group, a significant expansion was observed on day 15 compared to baseline levels (day 0 and day 15 *p* < 0.0001). By Day 15 and 30 p.i. activated IgG^+^ MBC levels reach similar frequencies between both groups, yet a strong trend is observed where the undepleted control cohort shows higher levels of activated IgG^+^ MBCs. This data suggests that a lack of CD4^+^ T cell priming promotes an early expansion of a polyclonal (most likely less affinity matured) IgG^+^ MBC response that can be correlated to the lower specific IgG anti-DENV2 and neutralizing response. In addition, the early significant polyclonal expansion associated to a limited priming by CD4^+^ T cells also relates to an increased binding breadth to DENV4 EDIII domain and to the NS1 protein, as well as cross-reactivity to ZIKV in the convalescent period in the CD4-depleted cohort, as previously shown.

### 3.10. CD4 T Cell Depletion Before DENV2 Infection Results in a Delay of Cytokine Profiles in Rhesus Macaques

We measured B cell activation factor (BAFF) levels on days 0, 7 and 10 post DENV2 infection (Figure S13). No differences between groups were observed. Serum cytokines were measured via Luminex assay pipeline using samples collected from days 0, 3 and 7 post DENV2 infection for all CD4-depleted and undepleted animals. Among the eleven measured cytokines, only three (IFN-alpha, BCA-1 and IP-10) had statistically significant differences (Figure S14). There were no significant differences between groups in any of the timepoints measured for IFN-alpha. By day 7, all animals had a significant increase of IFN-alpha when compared to day 0 and day 3 (CD4-depleted *p* = 0.0129 and undepleted *p* = 0.0023). Even though both groups show an increase in IFN alpha over time, undepleted animals produced more of this cytokine than the CD4-depleted group. This suggests that the lack of CD4 T cells may result in a delay of IFN alpha production (Figure S14A).

When analyzing BCA-1 (CXCL13), there were no statistically significant differences between groups in days 0 and 3. However, by day 7 CD4-depleted animals had significantly lower levels of BCA-1 than the undepleted group (*p* = 0.0312). Also, in the undepleted animals, BCA-1 levels significantly increased by day 7 compared to day 0 (*p* = 0.0234) (Figure S14B).

When comparing IP-10 (CXCL10) levels, there were no significant differences between groups at any timepoint. However, CD4-depleted animals had significantly higher levels of IP-10 by day 3 (*p* = 0.0240), which eventually increased more by day 7 (*p* < 0.0001) compared to their baseline levels. On the other hand, undepleted animals had significantly higher values of IP-10 on day 7 in comparison with their baseline levels (*p* = 0.0372). Surprisingly, although not statistically significant, the CD4-depleted group had higher levels of IP-10 in serum in comparison with the undepleted group by days 3 and 7 post DENV2 infection (Figure S14C). These results support the higher viral replication observed in the CD4-depleted animals, which in turn may suggest a higher production of IP-10 by monocytes and dendritic cells (DCs).

Interestingly, although no significant differences were observed, CD4-depleted animals had undetectable IFN-gamma levels in all three timepoints measured in comparison to the undepleted group, that had approximately half of the animals producing detectable levels of IFN-gamma. This implies that CD4 T cells may play a role inducing the production of IFN-gamma for viral clearance.

Overall, we observed an expected cytokine profile response in all individuals, with cytokine levels showing a trend to increase after days 3 and 7 post DENV2 infection compared to their baseline levels. In summary, CD4-depleted animals demonstrate a delayed response in almost all the cytokines measured compared to the undepleted animals (except for IFN-gamma profiles). Taking this into consideration, in general our results suggest that CD4 T cells may play a role in the induction of several cytokines and the activation of other immune cells necessary for viral clearance.

## 4. Discussion

Despite decades of research, dengue virus (DENV) pathogenesis remains incompletely understood. A balanced interaction between humoral and T cell responses is known to be crucial for protection. The role of CD4^+^ T cells in this balance has been revisited in recent years, shifting from an early focus on pathogenic contributions to a more nuanced appreciation of their protective functions [55,56]. Recent studies underscore their importance in viral clearance, B cell priming, and modulation of antibody quality and specificity.

Our findings, derived from a rhesus macaque model of primary DENV2 infection, demonstrate that CD4^+^ T cells play a central role in shaping the early immune response. CD4^+^ T cell-depleted animals exhibited prolonged viremia, elevated NS1 antigenemia, delayed class switching, and increased frequencies of cross-reactive but weakly neutralizing antibodies. These data support a model in which CD4^+^ T cells are critical for efficient viral clearance and for promoting an antibody repertoire with greater neutralization capacity and reduced potential for antibody-dependent enhancement (ADE).Remarkably, CD4^+^ T cell-depleted macaques developed an altered humoral landscape characterized by early polyclonal activation of naïve and memory B cell compartments, diminished virus-specific IgM and IgG responses at early time points, and a delayed emergence of neutralizing antibodies against DENV2 and DENV4. Additionally, a predominance of IgG1 subclass was observed, with a notable lack of IgG2 detection. While the kinetics of IgG subclasses can have a significant effect on either disease severity and/or Ab response neutralization, various human and NHP studies have demonstrated a trend of IgG1 selection over IgG2 and other subclasses during DENV infections [57,58,59,60]. In the case of NHPs, IgG1 in macaques has been shown to induce both antibody-dependent cell cytotoxicity (ADCC) and phagocytosis, compared to the IgG2 subclass [61,62,63]. In addition, one past study showed that during DENV vaccination and challenge, IgG1 was the predominant subclass in macaques with higher Ab response neutralization [64]. Our work further shows that not only is the IgG1 response delayed in animals lacking CD4^+^ T cell priming, but also, we demonstrate an important role in this selection in IgG1, as the undepleted group showed better early control of viremia and higher neutralization compared to the CD4^+^ T cell-depleted cohort. In spite of this, by day 90 p.i., this pattern is reversed, and CD4-depleted animals show higher neutralizing activity, suggesting a potentially delayed but compensatory effect of antibody functionality in the absence of early CD4^+^ T cell help.

These functional deficits were mirrored by elevated levels of anti-NS1 IgG, which persisted one-year post-infection. Collectively, these findings point to a pivotal role for CD4^+^ T cells in supporting germinal center (GC) formation, affinity maturation, and durable, high-quality antibody production during primary DENV infection.

Interestingly, our observations in non-human primates differ from some murine studies. Yauch et al. (2010) reported that CD4^+^ T cell depletion did not impair early DENV-specific IgG or IgM titers or neutralization capacity in mice [65]. However, other murine studies have affirmed the protective role of CD4^+^ T cells in both vaccine-induced and natural infections, showing that depletion of these cells can abrogate vaccine-mediated protection and increase morbidity [66,67,68]. These discrepancies may reflect species-specific differences in immune architecture or the relative robustness of T cell-independent antibody responses in mice compared to primates.

Our findings align more closely with human data. For instance, children protected from symptomatic dengue have been shown to generate more robust anti-envelope (ENV) and anti-NS1 IgG with greater Fc effector function than symptomatic individuals [69]. Given recent efforts to include NS1 antigens in vaccine formulations, our findings suggest that NS1-specific responses may be qualitatively altered in the absence of CD4^+^ T cell help, potentially influencing durability and specificity. Our finding is supported by prior documentation that, like in humans, NS1 is an immunodominant target for CD4^+^ T cells in rhesus macaques [70,71].

Notably, our study highlights enhanced cross-reactivity to ZIKV NS1 in CD4^+^ T cell-depleted macaques—a finding rarely observed in primary DENV infection of flavivirus-naïve humans. This is supported by the presence of an antibody repertoire in depleted animals with increased binding to heterologous serotypes, particularly DENV1 and 3, but with limited or absent neutralizing activity, resembling the same type of Abs that induce ADE [25,72,73]. This suggests that CD4^+^ T cells may restrict the breadth of B cell responses to favor type-specific rather than broadly cross-reactive targets with limited functionality and underscore the role of those cells in averting ADE.

Overall, for all four serotypes of DENV, animals that lacked CD4 T cells had higher levels of binding antibodies than control animals in the linear portion of the curve.

This noteworthy finding strongly suggests that CD4^+^ T cells impact the antibody repertoire, against both autologous and heterologous serotypes. However, the extension of the impact may be serotype-related as the Abs properties are different for DENV1 and 3 compared to the heterologous DENV4 and autologous infecting DENV2. The differences in the Abs properties depending on the DENV serotype have been well-documented for sequential DENV-DENV infections [74,75] and, more recently, for DENV-ZIKV or ZIKV-DENV infections [76,77]. Our data adds new insight on a potential mechanism supporting those prior findings, suggesting the CD4^+^ T cells may be critical for the imbalance of the antibody’s repertoire against DENV serotypes and closely related flaviviruses.

While early neutralizing activity was significantly lower in the depleted animals, by day 300 p.i., neutralization against DENV2 and DENV4 surpassed that of controls. We can surmise that a potentially delayed affinity maturation is taking place for the B cell clones targeting the infecting DENV2 and to some degree, they cross-neutralize DENV4, where an effective and significantly higher neutralization at late timepoints p.i. is observed. Yet, the higher binding observed against DENVs 1 and 3 without an effective neutralization may represent a compensatory overshoot with a greater quantity of non-neutralizing polyclonal B cell clones with higher cross-binding. This adds to our claim on the importance of CD4^+^ T cell vaccine-induced priming, as the occurrence of such cross-binding clones at higher quantities may prove to be of lower efficacy to control subsequent infections in the real world and potentially promote detrimental effects with severe cases. A similar scenario was seen with persistence of anti-NS1 antibodies detected in the depleted animals almost 1 year after the primary DENV2 infection. The persistence of those Abs may also indicate a defect in establishing long-lived B cell memory and long-lived plasma cells (LLPCs). As consequence, instead of maintaining stable antibody titers, the response in depleted animals is more “noisy” and less regulated [78]. While there is limited work on the effect of CD4 T cells on anti-NS1 Abs dynamics, in a DENV mouse model, CD4-deficient animals showed markedly reduced neutralizing antibody and memory formation [65]. In a mouse model of ZIKV infection, the authors demonstrated that CD4^+^ deficiency reduces GC B cells, impairs LLPC formation, and leads to a rapid decline in neutralizing antibodies [79].

Mechanistically, we propose that the ratio of germinal center versus extrafollicular B cells is altered in the context of CD4^+^ T cell depleted, DENV-infected animals, leading to increased long-term neutralizing antibody responses. This way, in the context of a limited or delayed CD4 T cells engagement, as a consequence of a DENV vaccine design or a mild natural infection with limited specific epitopes presentation, the EF and GC responses may not be mutually exclusive but dynamically regulated [80,81]. This is evidenced by the early expansion of activated IgG^+^ memory B cells and naive/unclass-switched memory B cells in the CD4-depleted group—an expansion likely driven by T cell-independent activation pathways, possibly mediated through innate immune signals, such as BAFF or pattern recognition receptor (PRR) signaling. Similar polyclonal B cell activation has been described in both HIV and DENV infections and has been linked to poor antibody quality and affinity maturation [82,83,84,85]. Also, it is worth considering that delayed GC responses or T cell reconstitution over time may allow for eventual affinity maturation and functional antibody development. Similar kinetics have been observed in CD4-depleted murine models’ post-vaccination where authors showed the temporal flexibility of CD4^+^ T cell help [81]. That work showed that CD4^+^ T cell assistance does not need to occur at the moment of immunization. Help provided later, after CD4^+^ T cell recovery, as it happens in our NHP model, is sufficient for generating effective antibody responses, indicating a more dynamic window of CD4-dependent B cell support than previously appreciated.

Furthermore, from our results, we postulate that the properties and functionality of the increased long-term neutralizing antibody responses, in the absence or limited priming of CD4^+^ T cells, may be different depending on the antigenic uniqueness of each DENV serotype [86,87].

Although the cytokine analysis did not reveal dramatic group differences, a delayed interferon-alpha (IFN-α) and BCA-1 (CXCL13) response in CD4-depleted animals suggests a slower or impaired recruitment of immune cells necessary for GC responses. Interestingly, IP-10 (CXCL10), typically associated with monocyte and dendritic cell activation, was elevated in the CD4-depleted group, possibly reflecting compensatory innate activation in the face of uncontrolled viral replication.

These findings offer new insights into vaccine design. Most current DENV vaccines aim to elicit balanced neutralizing responses to all four serotypes. However, vaccine-induced antibodies often target cross-reactive, non-neutralizing epitopes, increasing the risk for ADE. Our data suggest that robust CD4^+^ T cell help—most likely from Tfh cells targeting ENV and NS1—may skew the response toward protective, type-specific neutralizing antibodies while avoiding the generation of broad but potentially pathogenic cross-reactivity. The imbalance of replication among serotype components, as observed in Dengvaxia and TAK-003 vaccines, may limit CD4^+^ T cell help for some serotypes, compromising protection and increasing risk during secondary dengue infections.

Importantly, this study highlights the translational value of the rhesus macaque model in flavivirus research. The immune architecture and GC biology of NHPs more closely mirror that of humans than murine models, offering unique insights into B cell priming, memory formation, and antibody evolution in vivo.

## 5. Conclusions

Altogether, our findings reinforce the central role of CD4^+^ T cells in promoting timely, high-quality, type-specific antibody responses during primary DENV infection. Their absence leads to delayed class switching, polyclonal memory B cell activation, enhanced antibody cross-reactivity—particularly to NS1—and increased potential for ADE. These observations have direct implications for dengue and ZIKV vaccine development. Future vaccine strategies must incorporate CD4^+^ T cell epitopes—particularly from NS1 and envelope proteins—and ensure their effective presentation to drive protective humoral immunity. An additional layer of complexity is added by the differences in the CD4^+^ T cells epitopes dominance among flavivirus in general [88,89] and among DENV serotypes [86,87]. Moreover, vaccine-induced memory should aim to mimic the protective imprinting observed in natural infection with intact CD4^+^ T cell support as it has been pointed out before [90], to mitigate the risk of cross-reactive pathology upon secondary flavivirus exposure. The consequences of these depletions during a primary infection on a secondary heterologous infection (by another DENV serotype or other related flavivirus) remain to be investigated. However, we can anticipate that the limited imprinted immune response may have functional consequences in the resolution and outcome of a secondary infection. Our results strengthen the complexity of immunological interactions among DENV-serotypes and particularly underscore the pleomorphism of DENV immunology when it comes to the underestimated paradox of CD4^+^ T cells dependent versus independent pathways.

## 6. Limitations

While this study provides mechanistic insights into the role of CD4^+^ T cells during primary DENV2 infection, several limitations should be acknowledged. First, rhesus macaques, though immunologically similar to humans, do not fully recapitulate the clinical spectrum of dengue disease, which is typically mild or subclinical in non-human primates. Second, while we observed significant alterations in the humoral response due to CD4^+^ T cell depletion, the specific clonality, affinity, and functional profiles of the resulting antibody repertoires were not assessed at the single-cell or BCR sequence level. Third, peripheral T follicular helper (Tfh) cells were not directly characterized due to CD4^+^ T cell depletion, limiting our ability to dissect the cellular mechanisms underlying altered B cell help. Fourth, by definition, the term ADE requires the integration of both the data shown here, and complete clinical lab data suggestive of disease status. Since we did not include complete lab and clinical data, we regard our ADE findings as an increase in ZIKV replication. Finally, although cytokine analyses revealed delayed immune activation in CD4-depleted animals, the scope was limited to a predefined panel, and broader immune profiling could uncover additional pathways affected by CD4^+^ T cell loss. Future studies incorporating single-cell immunoprofiling and in-depth Tfh analysis will be essential to fully elucidate the cellular interactions governing flavivirus immunity.

## Figures and Tables

**Table 1:** DENV-2 RNAemia of CD4-depleted and undepleted animals.

ID	Immune History	RNAemia (Log10 Genome Copies/mL) Post-DENV Infection																	Days	
		0	1	2	3	4	5	6	7	8	9	10	11	12	13	14	15	30	Total	Mean
**0Z6**	**CD4-/DV2**	ND	2.905	3.924	4.505	4.245	3.875	3.799	3.040	ND	2.259	3.446	3.615	3.039	ND	ND	ND	ND	**117**	**9.75**
AE9		ND	2.834	4.204	4.759	4.059	3.758	3.164	3.545	2.957	2.540	2.623	3.272	3.202	ND	ND	ND	ND		
1Z3		ND	3.120	4.123	4.672	4.528	3.641	3.768	3.566	3.828	2.786	2.720	2.892	ND	ND	ND	ND	ND		
MA317		ND	2.682	4.557	5.235	4.888	4.291	3.176	ND	2.356	2.083	2.293	ND	2.492	ND	ND	ND	ND		
MA312		ND	2.493	3.908	5.060	3.718	3.961	3.217	3.425	3.285	3.107	3.713	ND	ND	ND	ND	ND	ND		
MA209		ND	2.167	3.323	4.003	3.844	2.765	4.283	4.608	4.784	4.765	4.202	3.555	3.018	ND	ND	ND	ND		
5Z1		ND	3.357	3.655	4.967	4.298	2.869	2.325	ND	ND	1.876	ND	ND	ND	ND	ND	ND	ND		
0Z7		ND	3.326	3.991	6.821	4.282	3.663	3.494	3.490	2.549	3.720	2.578	3.362	2.403	ND	ND	ND	ND		
MA333		ND	ND	4.111	4.812	4.456	3.692	3.147	3.429	2.831	2.604	2.368	2.600	ND	ND	ND	ND	ND		
MA321		ND	2.226	3.873	4.753	4.844	4.016	3.659	2.770	2.786	ND	2.294	1.945	ND	ND	ND	ND	ND		
MA267		ND	3.030	3.805	4.459	4.467	4.334	3.236	1.572	ND	2.916	1.257	2.786	ND	ND	ND	ND	ND		
MA316		ND	3.192	3.928	4.785	4.840	4.632	4.127	3.785	3.515	2.220	2.540	ND	ND	ND	ND	ND	ND		
9X5	**CD4+/DV2**	ND	3.076	3.879	4.741	4.379	3.663	2.551	ND	ND	ND	ND	ND	ND	ND	ND	ND	ND	**126**	**7.4**
MA341		ND	1.473	5.037	5.855	5.111	4.006	3.174	3.054	3.114	ND	ND	ND	ND	ND	ND	ND	ND		
4Z0		ND	2.466	4.092	4.865	4.364	3.085	3.052	3.474	2.524	2.003	ND	ND	ND	ND	ND	ND	ND		
MA320		ND	3.566	4.598	4.650	4.641	3.674	2.390	ND	2.401	ND	ND	ND	ND	ND	ND	ND	ND		
8X2		ND	3.598	4.078	4.505	3.255	1.983	3.257	2.227	ND	1.119	ND	ND	ND	ND	ND	ND	ND		
MA258		ND	1.853	3.330	4.924	4.692	4.249	3.768	3.313	1.457	ND	ND	ND	ND	ND	ND	ND	ND		
9X7		ND	2.895	4.247	5.148	4.305	2.716	1.266	ND	ND	ND	ND	ND	ND	ND	ND	ND	ND		
5Z8		ND	3.901	4.555	5.487	4.782	3.123	1.193	1.898	2.797	ND	ND	ND	ND	ND	ND	ND	ND		
9X2		ND	3.638	4.313	4.662	4.704	4.079	3.456	2.340	3.067	ND	ND	ND	ND	ND	ND	ND	ND		
9X1		ND	2.408	3.804	4.242	4.009	3.552	2.940	2.190	2.190	2.421	ND	ND	ND	ND	ND	ND	ND		
2Z4		ND	3.604	3.827	4.477	4.215	3.089	2.754	3.162	2.792	3.159	ND	ND	ND	ND	ND	ND	ND		
MA163		ND	3.103	3.762	4.026	4.581	4.030	4.078	3.911	3.282	3.605	ND	ND	ND	ND	ND	ND	ND		
MA254		ND	2.470	4.443	5.228	5.012	3.087	ND	ND	ND	ND	ND	ND	ND	ND	ND	ND	ND		
MA264		ND	2.087	4.210	3.857	5.160	4.333	3.097	ND	ND	ND	ND	ND	ND	ND	ND	ND	ND		
MA311		ND	3.243	4.216	4.511	4.387	2.845	2.551	ND	ND	ND	ND	ND	ND	ND	ND	ND	ND		
MA350		ND	1.630	4.534	5.385	5.190	3.504	1.688	1.183	2.971	ND	ND	ND	ND	ND	ND	ND	ND		
MA364		ND	2.779	4.229	5.033	5.035	4.128	3.654	ND	ND	1.884	ND	ND	ND	ND	ND	ND	ND		

DENV2 RNA detection during the first 15 days and day 30 p.i. Mean viremia days per group were calculated using days with detectable RNAemia divided by the number of animals in each group. ND = viral RNA not detected.

## Data Availability

All relevant data are given within the manuscript and its Supporting Information Files.

## Supplementary Materials

The following supporting information can be downloaded at: https://www.mdpi.com/article/10.3390/vaccines13111103/s1, Figure S1: CD4^+^ and CD8^+^ T cell frequency following depletion treatment before and after DENV2. Frequency of CD4^+^ and CD8^+^ T cells was assessed by immunophenotyping before and after depletion treatments using flow cytometry. CD4-depleted animals are depicted in purple and undepleted animals are depicted in turquoise; Figure S2: Gating strategy for T cells. Gating strategy used to define T cells and subsets. Lymphocytes were gated based on their characteristic forward and side scatter pattern (FSC, SSC). T cells were defined as CD3+CD20+. CD4^+^ and CD8^+^ T cells were defined as CD3+CD4+ and CD3+CD8+, respectively; Figure S3: Gating strategy for B cells. Gating strategy used to define B cells and subsets. Lymphocytes were gated based on their characteristic forward and side scatter pattern (FSC, SSC). B cells were defined as CD20+CD3-. Memory (MBC = CD20+, CD3-, CD27+), class-switched IgG Memory (IgG MBC = CD20+, CD3-, CD27+, surface IgG+ (sIgG)), and unclass switched memory (CD3-CD20+CD27-). Activated phenotypes were measured via the inclusion of the CD69+ marker; Figure S4: DENV NS1 levels are affected by absence of CD4^+^ T cells after DENV2 infection. DENV-NS1 levels after DENV-2 infection were measured using a commercial ELISA test. CD4- depleted animals are depicted in purple and undepleted animals are depicted in turquoise. Dotted lines indicate the limit of detection for each test. Statistically significant differences among and within groups were calculated by two-way ANOVA using Tukey’s multiple comparisons test and unpaired *t*-tests; Figure S5: Kinetics of hematology and laboratory results. Cell subsets obtained from complete blood count (CBC) and comprehensive metabolic panel (CMP) tests at pre-treament (Pre-Tx), baseline, and days 6, 10, 15, 30, 60 and 90 p.i. CD4-depleted animals are depicted in purple and undepleted animals are depicted in turquoise. Statistically significant differences among and within groups were calculated by two-way ANOVA using Tukey’s multiple comparisons test; Figure S6: T cell depletion modifies serological profile during primary DENV2 infection. IgM and IgG response after DENV-2 infection was assessed using commercial ELISA tests. CD4- depleted animals are depicted in purple and undepleted animals are depicted in turquoise. Dotted lines indicate the limit of detection for each test. (A) Binding capacity of DENV IgM and IgG antibodies from CD4-depleted and undepleted animals after DENV2 infection. (B) Total DENV IgM levels throughout different timepoints. (C) Binding capacity of DENV IgG antibodies from CD4-depleted and undepleted animals after DENV2 infection. (D) Total DENV IgG levels throughout different timepoints. Statistically significant differences among and within groups were calculated by two-way ANOVA using Tukey’s multiple comparisons test and unpaired *t*-tests; Figure S7: CD4^+^ T cell depletion modifies IgG subclass kinetics during primary DENV infection. IgG subclass (IgG1) levels were assessed in sera collected on days 0, 15, 30, 90, and 300 post DENV2 infection via in-house ELISA. CD4-depleted animals are depicted in purple, and undepleted animals are depicted in turquoise. Binding capacity of DENV IgG1 subclass antibodies in CD4^+^ T cell-depleted and undepleted animals after DENV2 infection in different timepoints is shown. Statistically significant differences among and within groups were calculated by two-way ANOVA using Tukey’s multiple comparisons test and unpaired *t*-tests; Figure S8: Depletion of CD4^+^ T cells before DENV infection increases DENV NS1 anti-IgG levels. DENV NS1 anti-IgG levels after DENV2 infection. CD4-depleted animals are depicted in purple and undepleted animals are depicted in turquoise. No limit of detection or cut-off value is provided because this value will vary depending on flavivirus disease prevalence in the geographical location where the test is performed. For this reason, cut-off value was set at 2 standard deviations of the average value from baseline data. Statistically significant differences among and within groups were calculated by two-way ANOVA using Tukey’s multiple comparisons test and multiple *t*-tests; Figure S9: Endpoint dilutions IgG binding ELISA against ZIKV. The quality of the IgG humoral immune response against ZIKV was accessed using an endpoint dilution binding Elisa. Serum of 300 days post primary DENV2 infection was used. CD4-depleted animals are depicted in purple and undepleted animals are depicted in turquoise. Statistical analysis among groups were observed using two-way ANOVA to compare the values of CD4-depleted DENV2 and undepleted groups; Figure S10: Lack of CD4^+^ T cells increases ZIKV cross-reactive antibody levels in DENV-immune animals. Binding capacity of ZIKV IgG antibodies from CD4-depleted and undepleted animals after DENV2 infection. CD4-depleted animals are depicted in purple and undepleted animals are depicted in turquoise. Dotted lines indicate the limit of detection for the test. Statistically significant differences among and within groups were calculated by two-way ANOVA using Tukey’s multiple comparisons test and unpaired *t*-tests; Figure S11: FRNT values of neutralizing antibodies against DENV and ZIKV in depleted and undepleted flavivirus-naïve macaques. FRNT60 values of neutralizing antibodies against DENV1, DENV3 and ZIKV are shown. CD4-depleted animals are depicted in purple and undepleted animals are depicted in turquoise. Dotted lines indicate the limit of detection for each test. Statistically significant differences among groups were calculated by one-way and two-way ANOVA using the Tukey’s multiple comparisons test and unpaired *t*-tests; Figure S12: Activation of naive and IgG+ memory B cell subset populations during a primary DENV2 infection in CD4^+^ T cell competent and -deficient animals. The frequencies of naive B cells and IgG+ MBCs were assessed by immunophenotyping using flow cytometry. CD4-depleted animals are depicted in purple and undepleted animals are depicted in turquoise. (A) Frequencies of naive B cells (CD3-CD20+CD27-) in total B cells (CD3-CD20+) after a primary DENV2 infection. (B) Frequencies of CD69+ cells in total naive B cell populations (defined as activated naive b cells CD3-CD20+CD27-CD69+). (C) Frequencies of total surface IgG expressing MBCs (CD3-CD20+CD27+sIgG+) in total MBCs. (D) Frequencies of CD69+ cells in total IgG+ MBCs (defined as activated IgG+ MBCs CD3-CD20+CD27+sIgG+CD69+). Statistical differences among groups were calculated by two-way ANOVA using Šidák’s and Turkey’s multiple comparisons test and paired multiple *t*-tests; Figure S13: BAFF protein levels in serum collected from Undepleted and CD4-Depleted animal cohorts after a primary DENV2 infection. BAFF levels were measured via ELISA in collected serum on days 0, 7, and 10 post-DENV2 infection. CD4-depleted animals are depicted in purple, and undepleted animals are depicted in turquoise. Dotted lines indicate the limit of detection of the kit. BAFF levels are depicted. Statistically significant differences among and within groups were calculated by two-way ANOVA using Tukey’s multiple comparisons test and unpaired *t*-tests; Figure S14: Serum cytokine responses during DENV-2 infection. Cytokine responses to DENV-2 infection were assessed via Luminex Assay using serum samples from baseline and days 3 and 7 post DENV2 infection. CD4-depleted animals are depicted in purple and undepleted animals are depicted in turquoise. Dotted lines indicate the limit of detection for each test. (A–K) All serum cytokines were measured in pg/mL. Statistically significant differences among and within groups were calculated by two-way ANOVA using Tukey’s multiple comparisons test and unpaired *t*-tests; All supplementary tables shown are for raw data. Table S1: Ab binding, BAFF, flow cytometry, and ADE raw data from the Undepleted and CD4-Depleted groups; Table S2: Raw data from clinical parameters surveyed include CMP and CBC. Table S3: ELISA raw data from Undepleted and CD4-Depleted cohorts; Table S4: Focus/Plaque Reduction Neutralization tests for DENV and ZIKV neutralization profiling raw data and cytokine profiling raw data from the Undepleted and CD4-Depleted cohorts.
