# Supplementary material for: CD4+ T Cells Are Key to Shaping a Protective Humoral Immunity in Primary Dengue 2 Virus Infection: Implications for Rational Vaccine Design
Source: Vaccines (Basel). 2025 Oct 29;13(11):1103. doi: 10.3390/vaccines13111103 (PMC12656844; doi:10.3390/vaccines13111103)
Supplement: Supplementary file 1 [file vaccines-13-01103-s001.zip › vaccines-3955435-supplementary.docx]

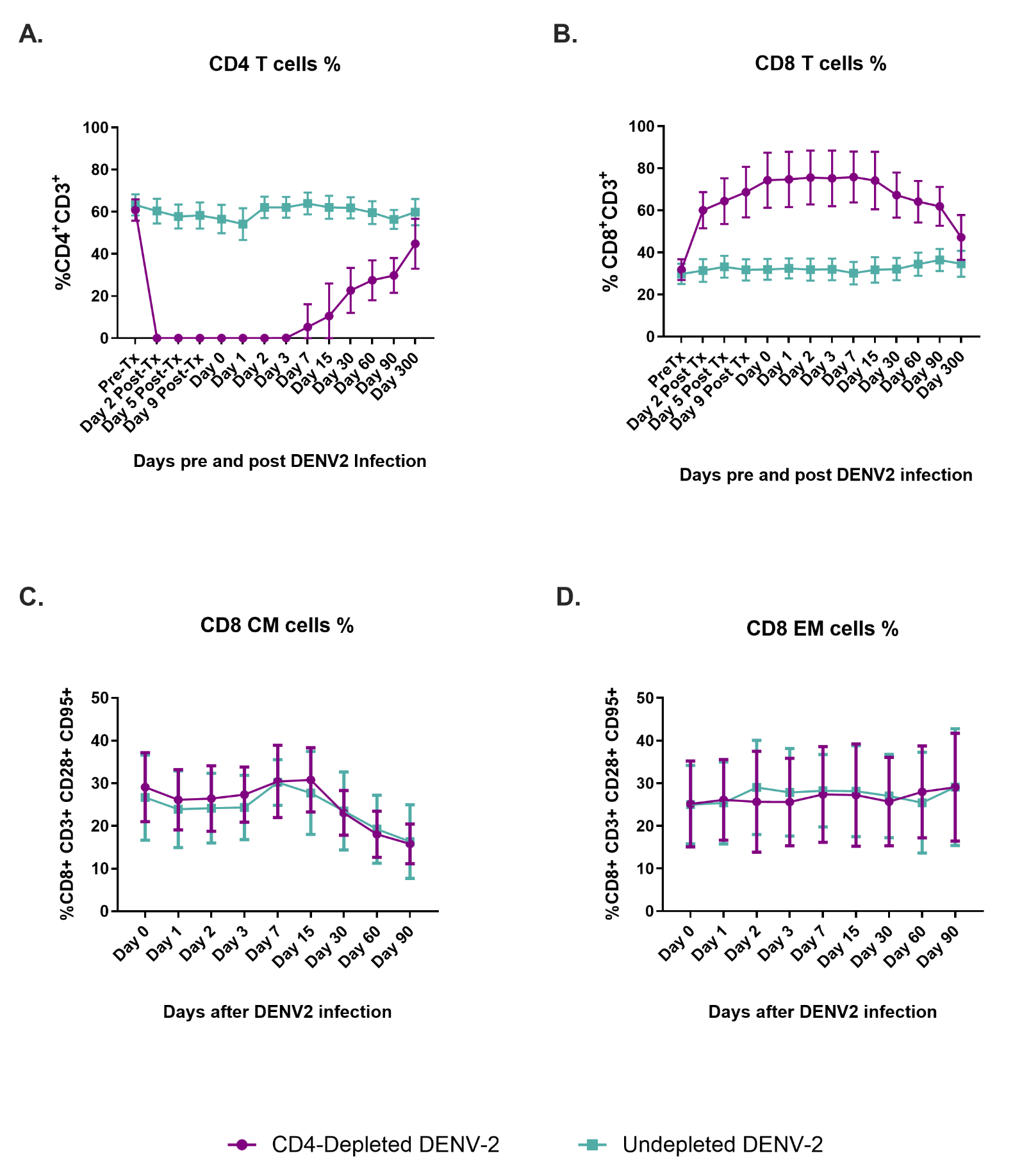


**Figure S1. CD4^+^ and CD8^+^ T cell frequency following depletion treatment before and after DENV2.** Frequency of CD4^+^ and CD8**^+^** T cells was assessed by immunophenotyping before and after depletion treatments using flow cytometry. CD4-depleted animals are depicted in purple and undepleted animals are depicted in turquoise.


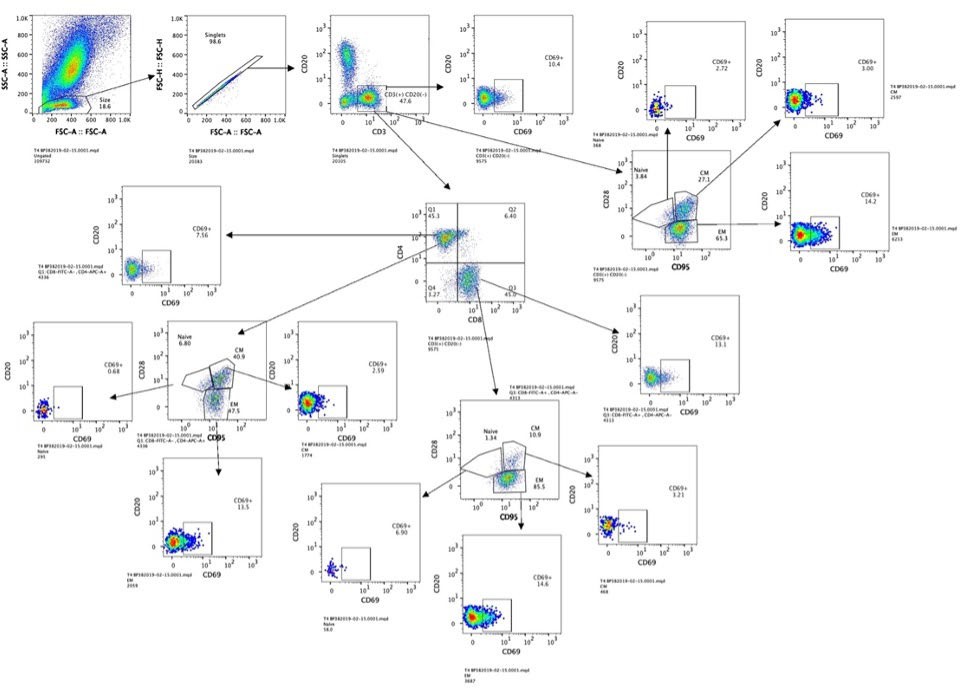


**Figure S2. Gating strategy for T cells.** Gating strategy used to define T cells and subsets. Lymphocytes were gated based on their characteristic forward and side scatter pattern (FSC, SSC). T cells were defined as CD3+CD20+. CD4+ and CD8+ T cells were defined as CD3+CD4+ and CD3+CD8+, respectively.


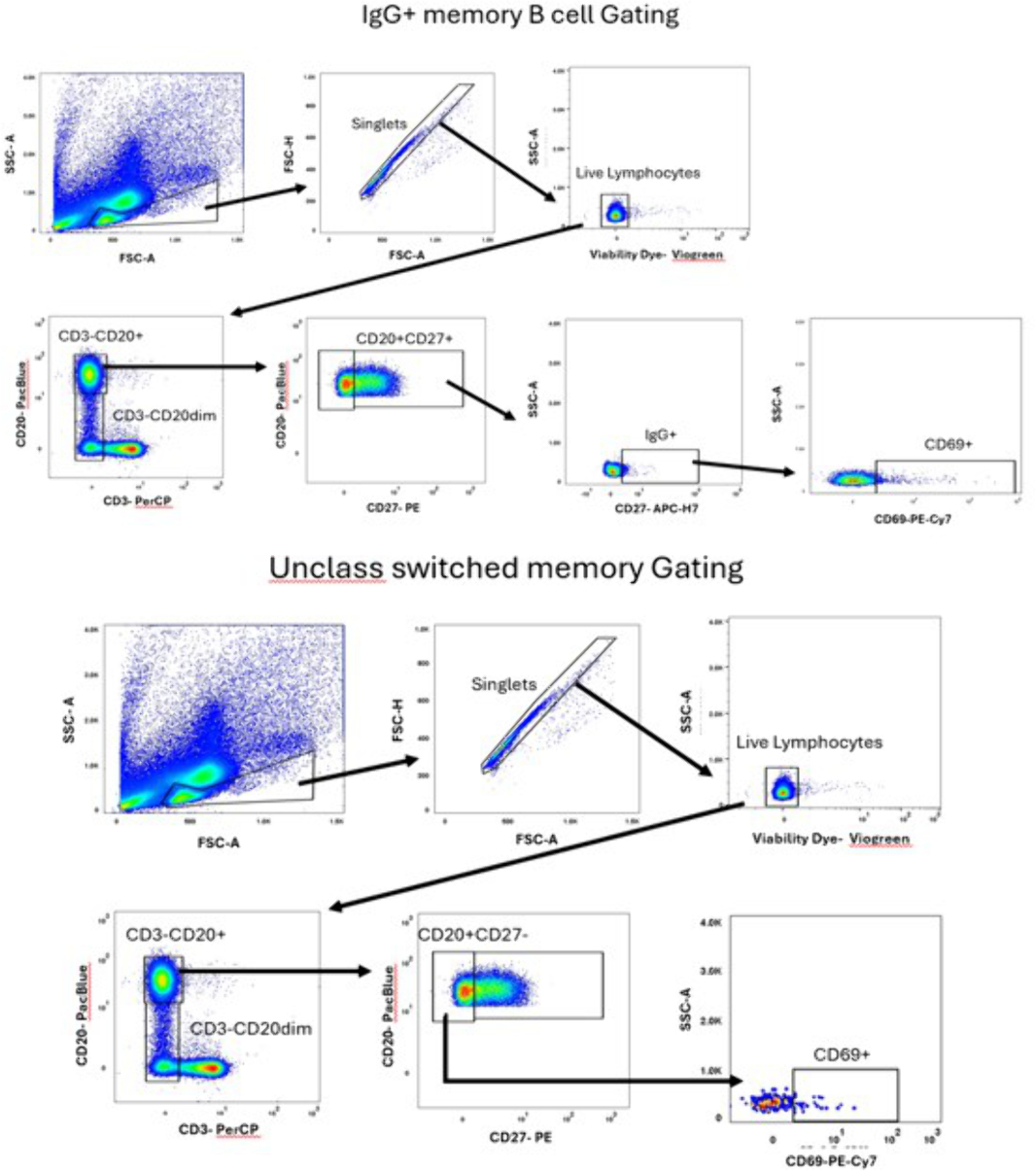


**Figure S3. Gating strategy for B cells.** Gating strategy used to define B cells and subsets. Lymphocytes were gated based on their characteristic forward and side scatter pattern (FSC, SSC). B cells were defined as CD20+CD3-. Memory (MBC= CD20+, CD3-, CD27+), class-switched IgG Memory (IgG MBC= CD20+, CD3-, CD27+, surface IgG+ (sIgG)), and unclass switched memory (CD3-CD20+CD27-). Activated phenotypes were measured via the inclusion of the CD69+ marker.


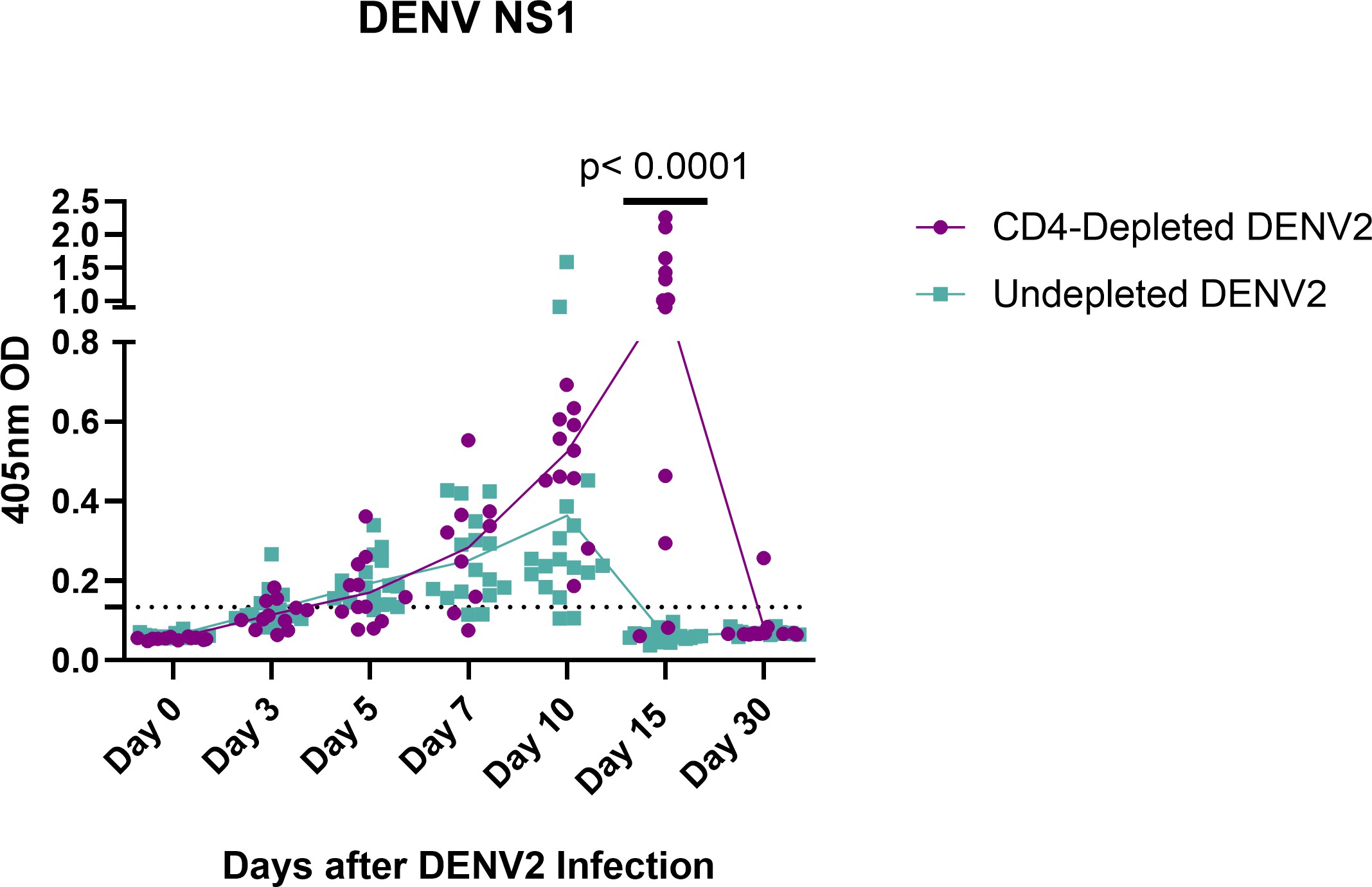


**Figure S4. DENV NS1 levels are affected by absence of CD4+ T cells after DENV2 infection.** DENV-NS1 levels after DENV-2 infection were measured using a commercial ELISA test. CD4- depleted animals are depicted in purple and undepleted animals are depicted in turquoise. Dotted lines indicate the limit of detection for each test. Statistically significant differences among and within groups were calculated by two-way ANOVA using Tukey’s multiple comparisons test and unpaired t-tests.


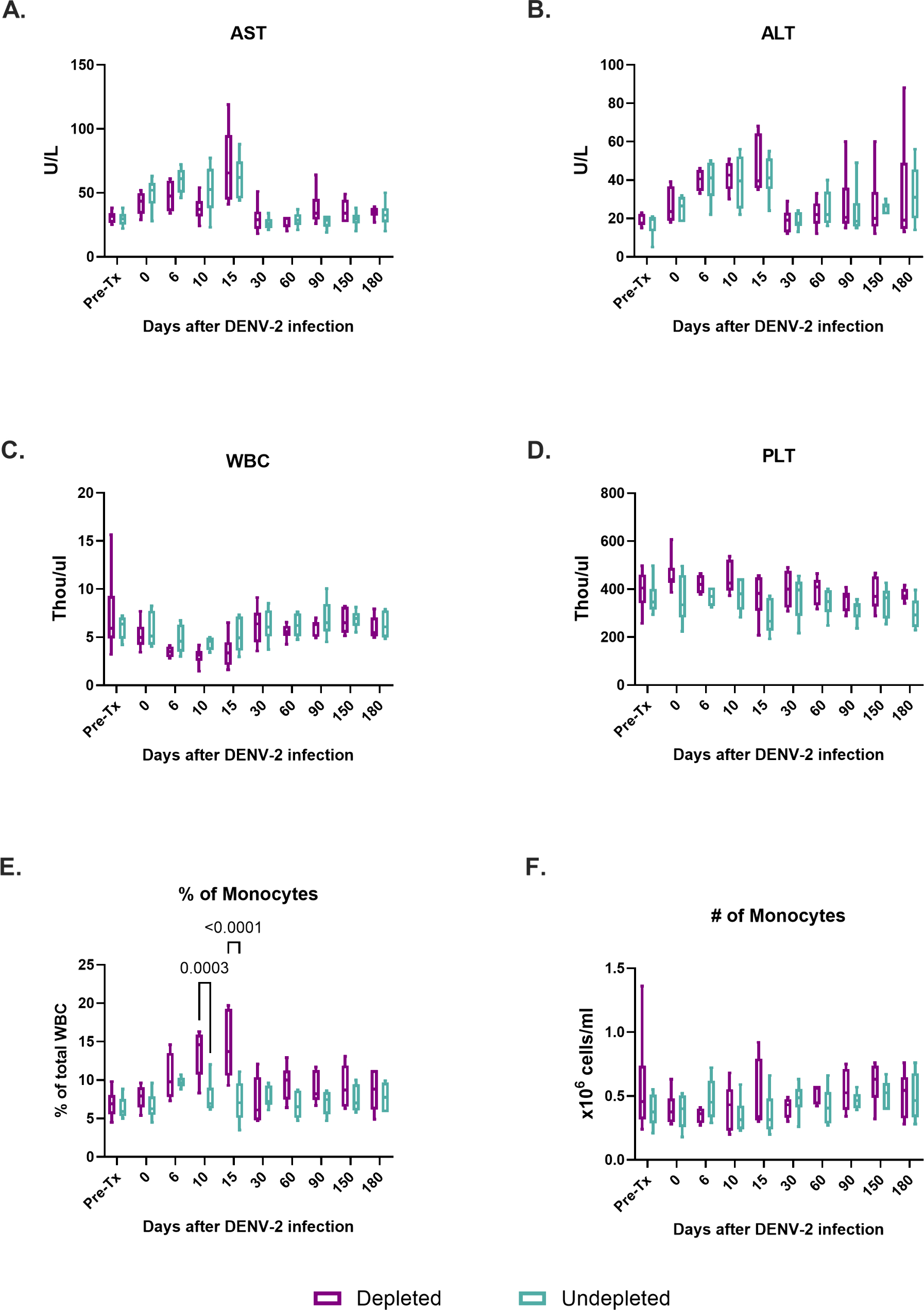


**Figure S5. Kinetics of hematology and laboratory results.** Cell subsets obtained from complete blood count (CBC) and comprehensive metabolic panel (CMP) tests at pre-treament (Pre-Tx), baseline, and days 6, 10, 15, 30, 60 and 90 p.i. CD4-depleted animals are depicted in purple and undepleted animals are depicted in turquoise. Statistically significant differences among and within groups were calculated by two-way ANOVA using Tukey’s multiple comparisons test.


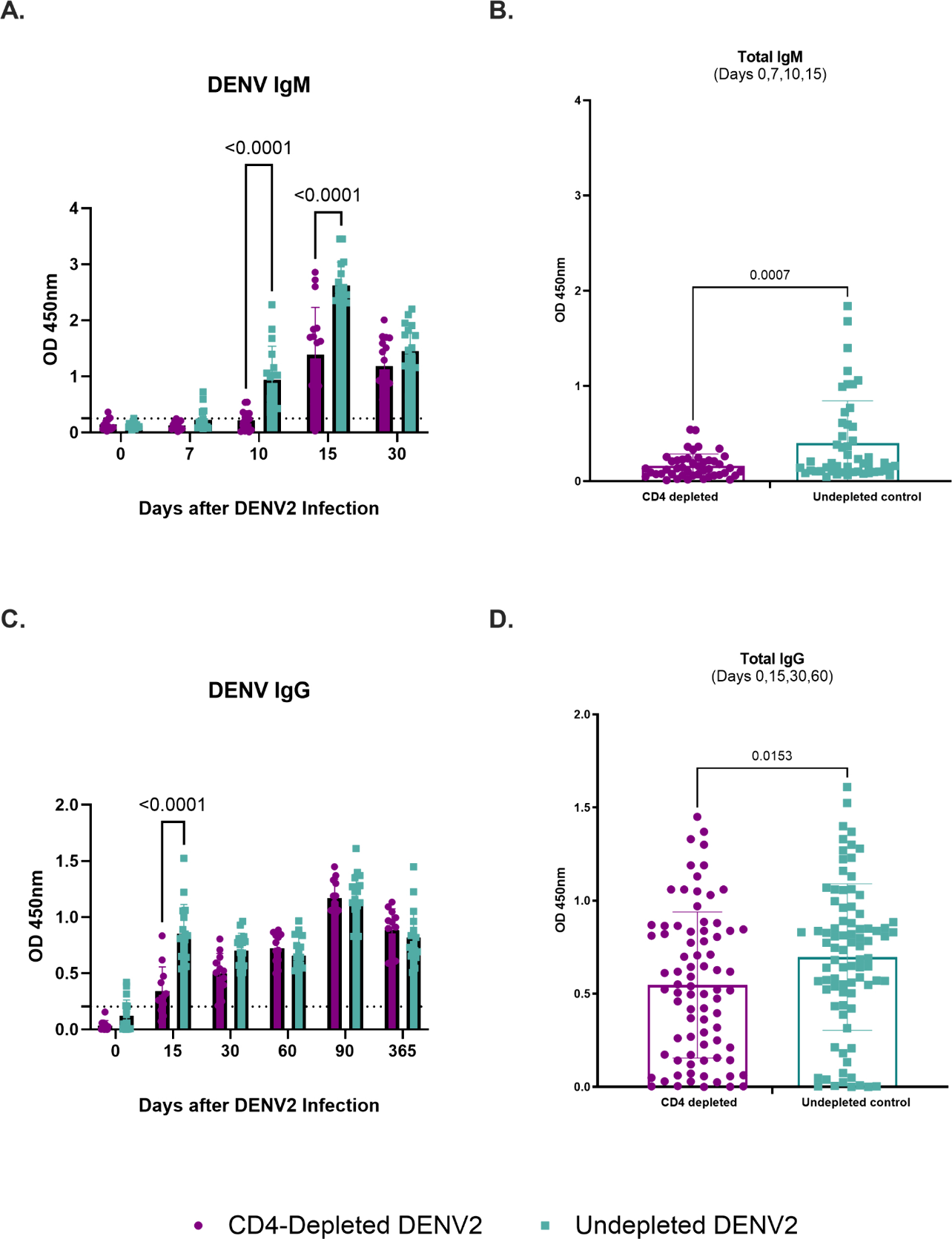


**Figure S6. T cell depletion modifies serological profile during primary DENV2 infection.** IgM and IgG response after DENV-2 infection was assessed using commercial ELISA tests. CD4- depleted animals are depicted in purple and undepleted animals are depicted in turquoise. Dotted lines indicate the limit of detection for each test. **(A)** Binding capacity of DENV IgM and IgG antibodies from CD4-depleted and undepleted animals after DENV2 infection. **(B)** Total DENV IgM levels throughout different timepoints. **(C)** Binding capacity of DENV IgG antibodies from CD4-depleted and undepleted animals after DENV2 infection. **(D)** Total DENV IgG levels throughout different timepoints. Statistically significant differences among and within groups were calculated by two-way ANOVA using Tukey’s multiple comparisons test and unpaired t-tests.


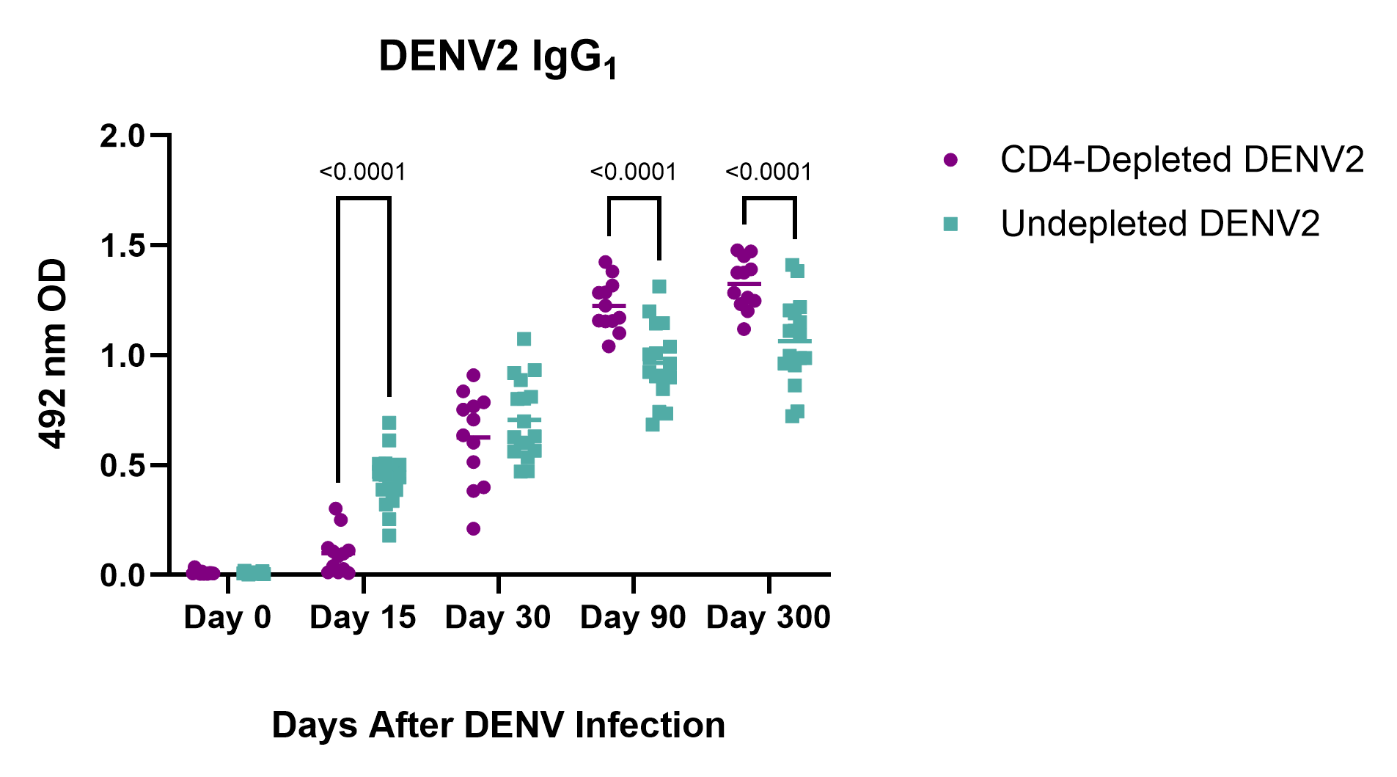


**Figure S7. CD4+ T cell depletion modifies IgG subclass kinetics during primary DENV infection.** IgG subclass (IgG1) levels were assessed in sera collected on days 0, 15, 30, 90, and 300 post DENV2 infection via in-house ELISA. CD4-depleted animals are depicted in purple, and undepleted animals are depicted in turquoise. Binding capacity of DENV IgG1 subclass antibodies in CD4+ T cell-depleted and undepleted animals after DENV2 infection in different timepoints is shown. Statistically significant differences among and within groups were calculated by two-way ANOVA using Tukey’s multiple comparisons test and unpaired t-tests.


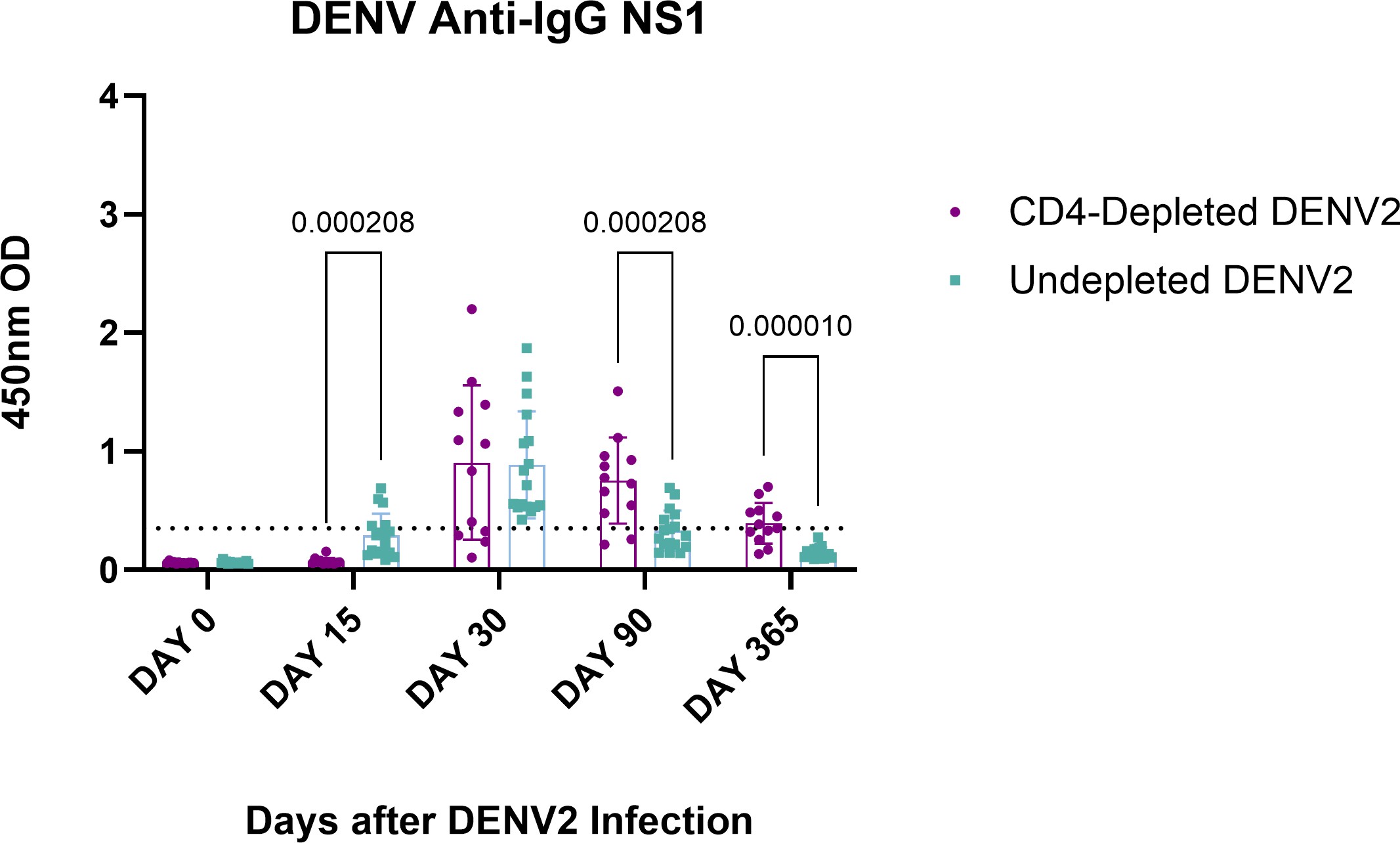


**Figure S8. Depletion of CD4+ T cells before DENV infection increases DENV NS1 anti-IgG levels.** DENV NS1 anti-IgG levels after DENV2 infection. CD4-depleted animals are depicted in purple and undepleted animals are depicted in turquoise. No limit of detection or cut-off value is provided because this value will vary depending on flavivirus disease prevalence in the geographical location where the test is performed. For this reason, cut-off value was set at 2 standard deviations of the average value from baseline data. Statistically significant differences among and within groups were calculated by two-way ANOVA using Tukey’s multiple comparisons test and multiple t-tests.


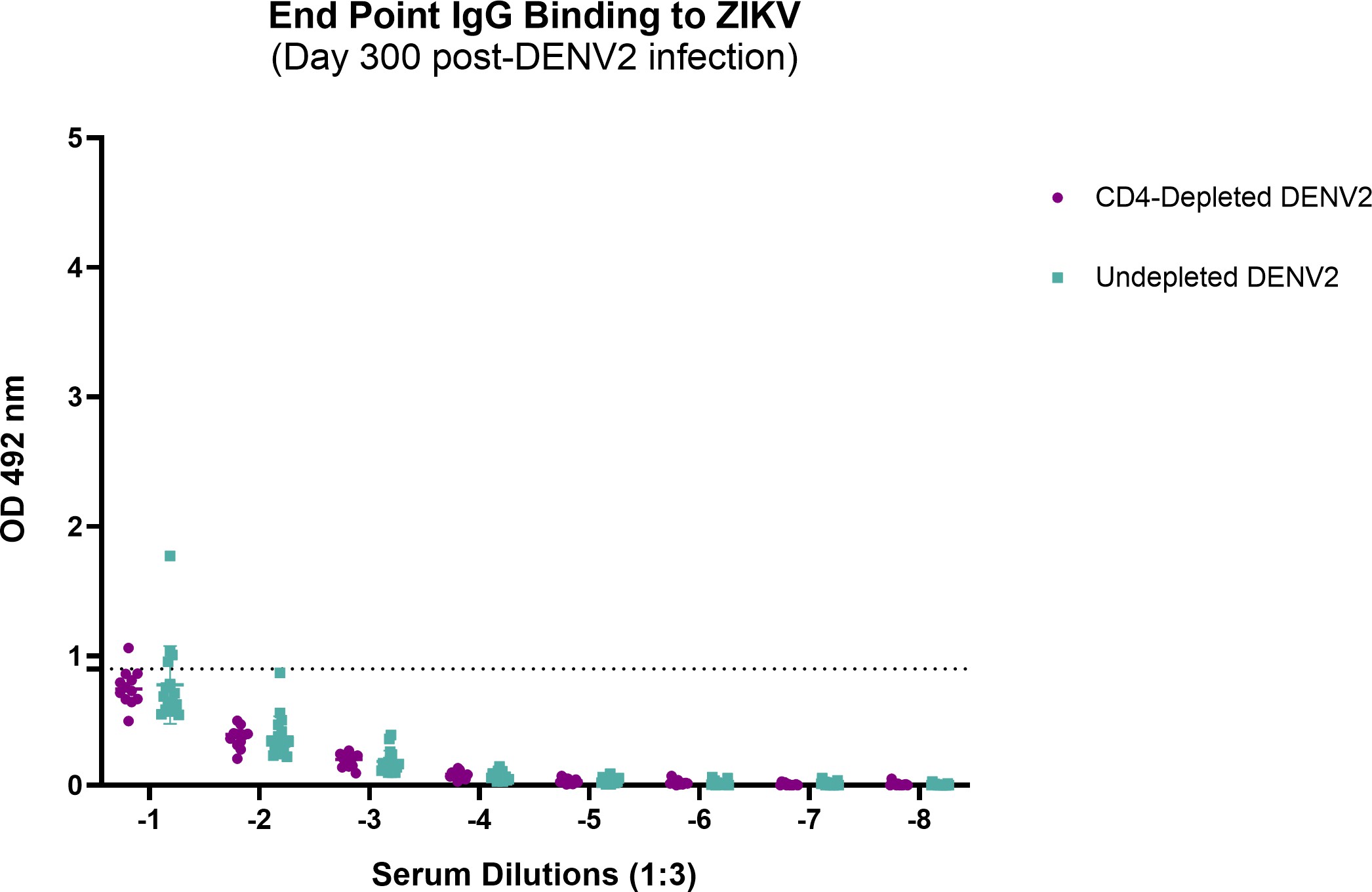


**Figure S9. Endpoint dilutions IgG binding ELISA against ZIKV.** The quality of the IgG humoral immune response against ZIKV was accessed using an endpoint dilution binding Elisa. Serum of 300 days post primary DENV2 infection was used. CD4-depleted animals are depicted in purple and undepleted animals are depicted in turquoise. Statistical analysis among groups were observed using two-way ANOVA to compare the values of CD4-depleted DENV2 and undepleted groups.


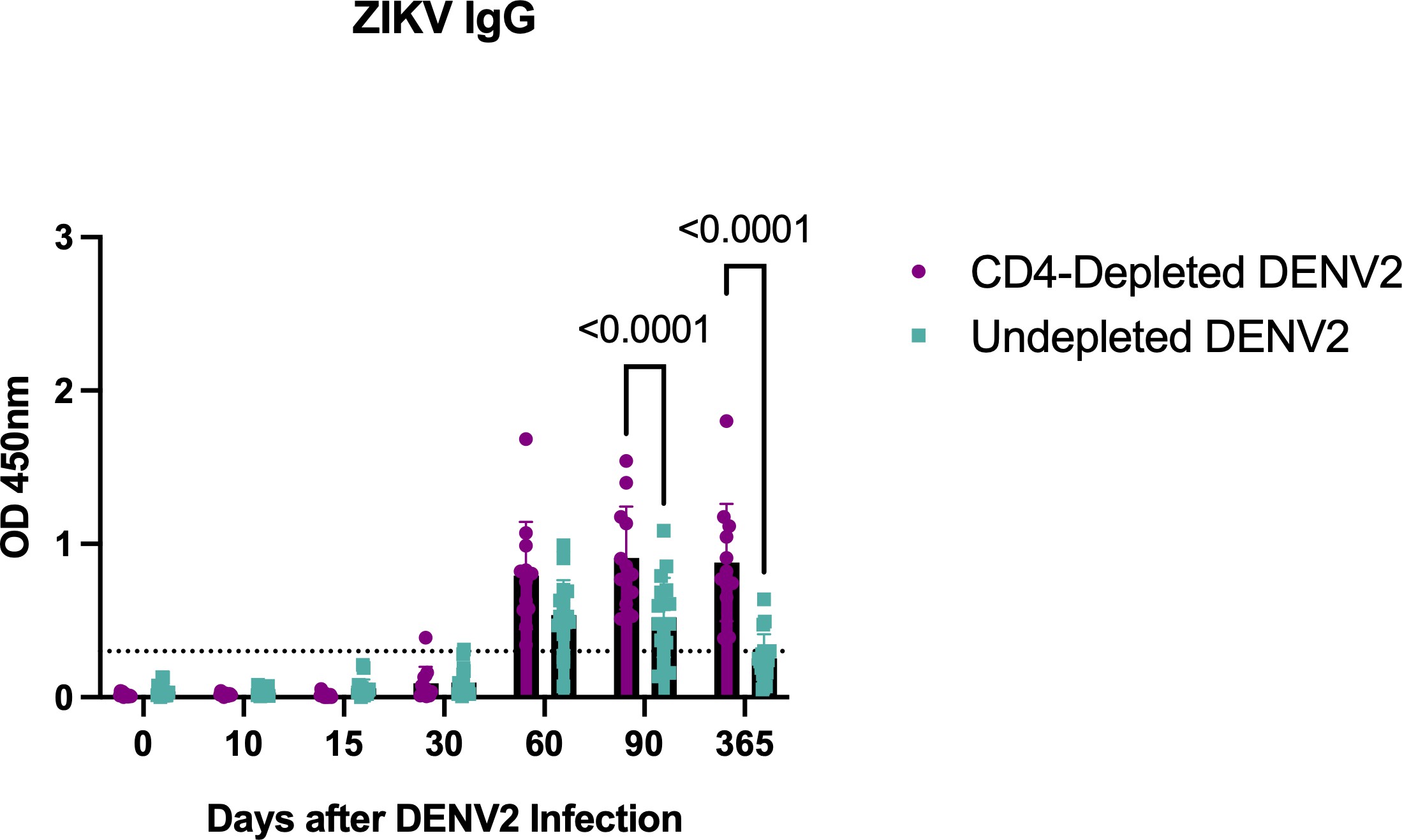


**Figure S10. Lack of CD4+ T cells increases ZIKV cross-reactive antibody levels in DENV-immune animals.** Binding capacity of ZIKV IgG antibodies from CD4-depleted and undepleted animals after DENV2 infection. CD4-depleted animals are depicted in purple and undepleted animals are depicted in turquoise. Dotted lines indicate the limit of detection for the test. Statistically significant differences among and within groups were calculated by two-way ANOVA using Tukey’s multiple comparisons test and unpaired t-tests.


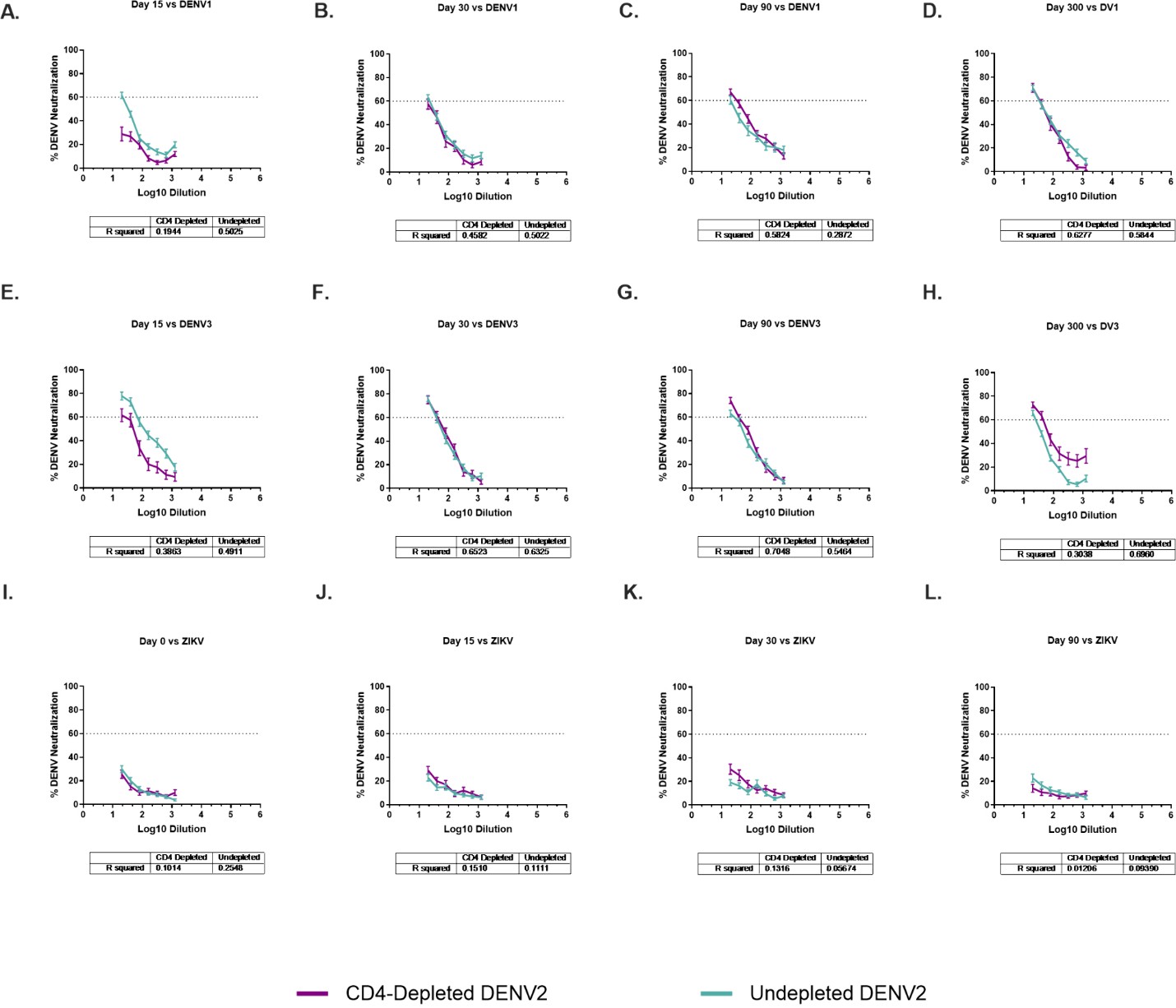


**Figure S11. FRNT values of neutralizing antibodies against DENV and ZIKV in depleted and undepleted flavivirus-naïve macaques.** FRNT60 values of neutralizing antibodies against DENV1, DENV3 and ZIKV are shown. CD4-depleted animals are depicted in purple and undepleted animals are depicted in turquoise. Dotted lines indicate the limit of detection for each test. Statistically significant differences among groups were calculated by one-way and two-way ANOVA using the Tukey’s multiple comparisons test and unpaired t-tests.


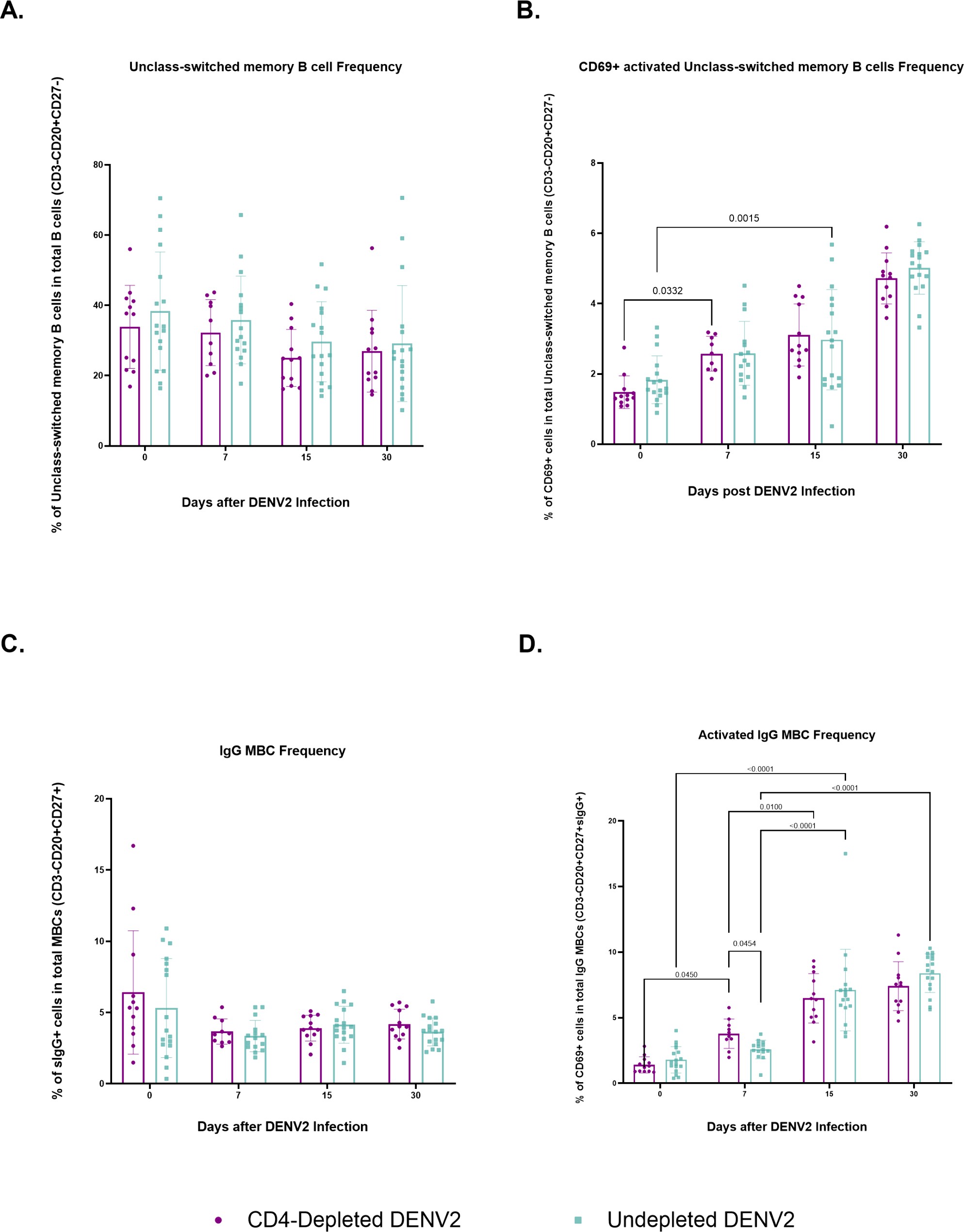


**Figure S12. Activation of naive and IgG+ memory B cell subset populations during a primary DENV2 infection in CD4+ T cell competent and -deficient animals.** The frequencies of naive B cells and IgG+ MBCs were assessed by immunophenotyping using flow cytometry. CD4-

depleted animals are depicted in purple and undepleted animals are depicted in turquoise. **(A)** Frequencies of naive B cells (CD3-CD20+CD27-) in total B cells (CD3-CD20+) after a primary DENV2 infection. **(B)** Frequencies of CD69+ cells in total naive B cell populations (defined as activated naive b cells CD3-CD20+CD27-CD69+). **(C)** Frequencies of total surface IgG expressing MBCs (CD3-CD20+CD27+sIgG+) in total MBCs. **(D)** Frequencies of CD69+ cells in total IgG+ MBCs (defined as activated IgG+ MBCs CD3-CD20+CD27+sIgG+CD69+). Statistical differences among groups were calculated by two-way ANOVA using Šidák’s and Turkey’s multiple comparisons test and paired multiple t- tests.


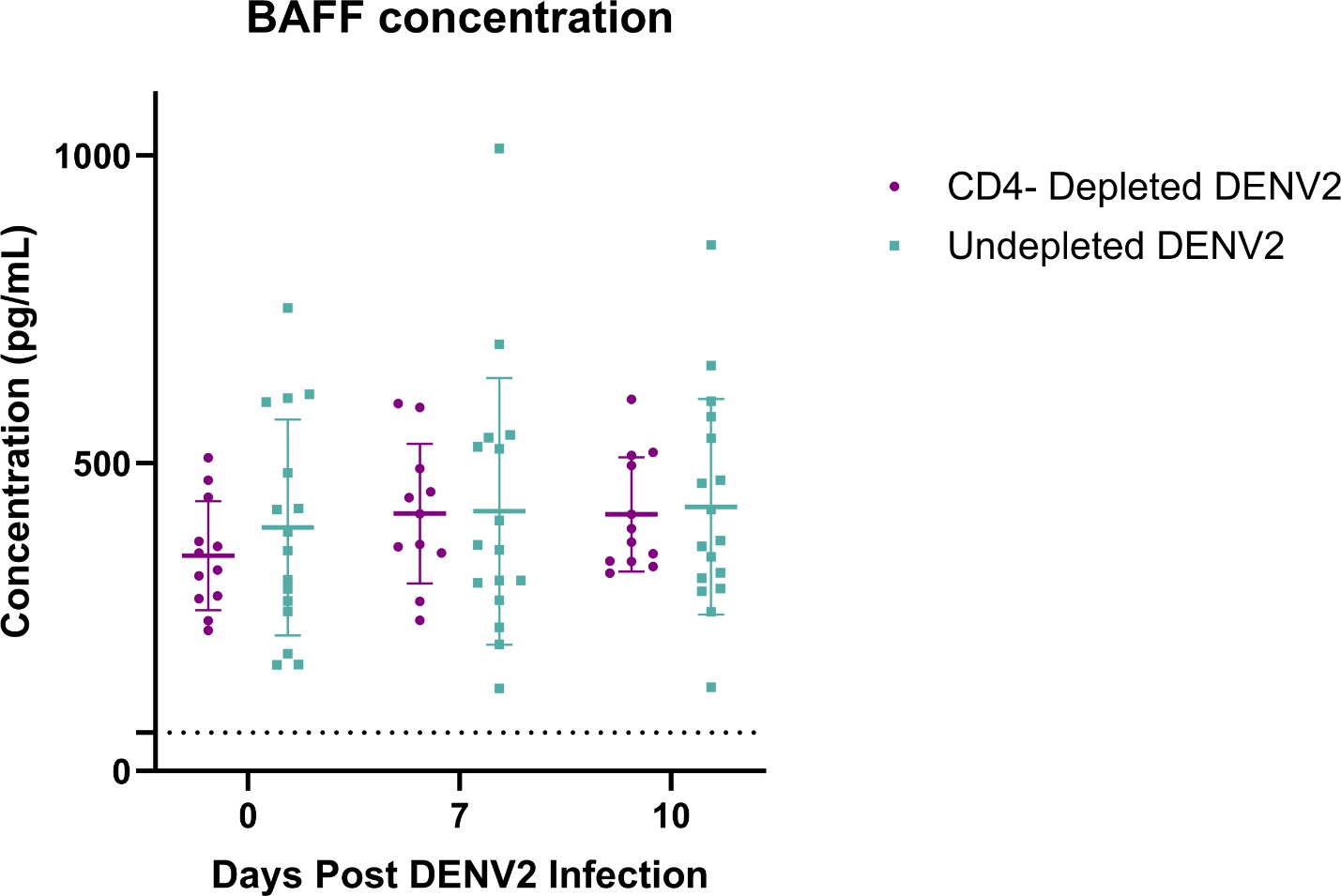


**Figure S13. BAFF protein levels in serum collected from Undepleted and CD4-Depleted animal cohorts after a primary DENV2 infection.** BAFF levels were measured via ELISA in collected serum on days 0, 7, and 10 post-DENV2 infection. CD4-depleted animals are depicted in purple, and undepleted animals are depicted in turquoise. Dotted lines indicate the limit of detection of the kit. BAFF levels are depicted. Statistically significant differences among and within groups were calculated by two-way ANOVA using Tukey’s multiple comparisons test and unpaired t-tests.


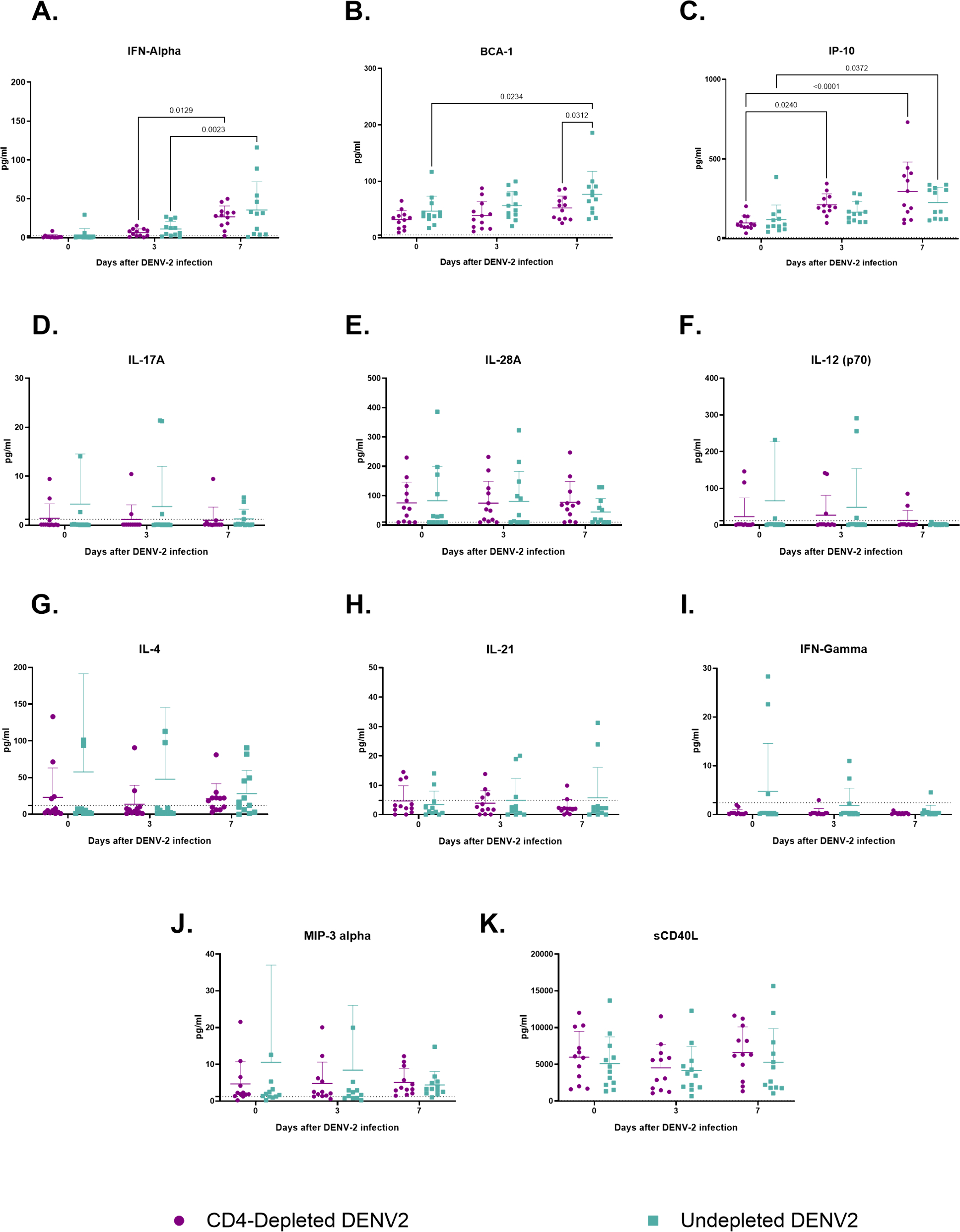


**Figure S14. Serum cytokine responses during DENV-2 infection.** Cytokine responses to DENV-2 infection were assessed via Luminex Assay using serum samples from baseline and days 3 and 7 post DENV2 infection. CD4-depleted animals are depicted in purple and undepleted animals are depicted in turquoise. Dotted lines indicate the limit of detection for each test. (A-K) All serum cytokines were measured in pg/ml. Statistically significant differences among and within groups were calculated by two-way ANOVA using Tukey’s multiple comparisons test and unpaired t-tests.
